# Exciton-harvesting enabled efficient charged particle detection in zero-dimensional halides

**DOI:** 10.1038/s41377-024-01532-z

**Published:** 2024-08-14

**Authors:** Qian Wang, Chenger Wang, Hongliang Shi, Jie Chen, Junye Yang, Alena Beitlerova, Romana Kucerkova, Zhengyang Zhou, Yunyun Li, Martin Nikl, Xilei Sun, Xiaoping OuYang, Yuntao Wu

**Affiliations:** 1 Shanghai Institute of Ceramics, Chinese Academy of Sciences, Shanghai, 201899 China; 2 Institute of High Energy Physics, Chinese Academy of Sciences, Beijing, 100049 China; 3 National Engineering Research Center for Rare Earth, Grirem Advanced Materials Co., Ltd. and General Research Institute for Nonferrous Metals, Beijing, 100088 China; 4 Department of Physics, Beihang University, Beijing, 100191 China; 5 Spallation Neutron Source Science Center, Dongguan, 523803 China; 6 Department of Optical Materials, Institute of Physics of the Czech Academy of Sciences, Prague, 16200 Czech Republic; 7 Northwest Institute of Nuclear Technology, Xi’an, 710024 China

**Keywords:** Metamaterials, Optical sensors

## Abstract

Materials for radiation detection are critically important and urgently demanded in diverse fields, starting from fundamental scientific research to medical diagnostics, homeland security, and environmental monitoring. Low-dimensional halides (LDHs) exhibiting efficient self-trapped exciton (STE) emission with high photoluminescence quantum yield (PLQY) have recently shown a great potential as scintillators. However, an overlooked issue of exciton-exciton interaction in LDHs under ionizing radiation hinders the broadening of its radiation detection applications. Here, we demonstrate an exceptional enhancement of exciton-harvesting efficiency in zero-dimensional (0D) Cs_3_Cu_2_I_5_:Tl halide single crystals by forming strongly localized Tl-bound excitons. Because of the suppression of non-radiative exciton-exciton interaction, an excellent α/β pulse-shape-discrimination (PSD) figure-of-merit (FoM) factor of 2.64, a superior rejection ratio of 10^−9^, and a high scintillation yield of 26 000 photons MeV^−1^ under 5.49 MeV α-ray are achieved in Cs_3_Cu_2_I_5_:Tl single crystals, outperforming the commercial ZnS:Ag/PVT composites for charged particle detection applications. Furthermore, a radiation detector prototype based on Cs_3_Cu_2_I_5_:Tl single crystal demonstrates the capability of identifying radioactive ^220^Rn gas for environmental radiation monitoring applications. We believe that the exciton-harvesting strategy proposed here can greatly boost the applications of LDHs materials.

## Abstract (web summary)

We propose and demonstrate an exciton-harvesting strategy in low-dimensional halides exposed to ionizing radiation, which enhances both scintillation efficiency and charged particle discrimination capability.

## Graphical abstract

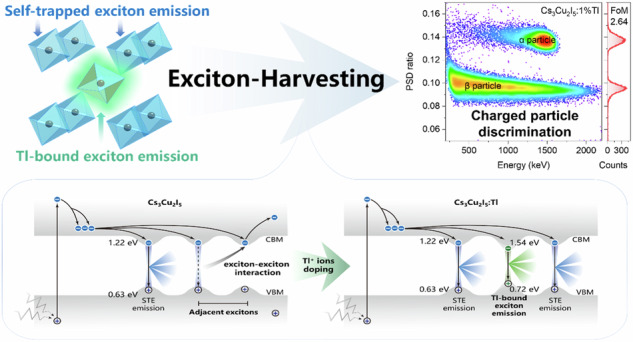

## Introduction

Radioactive element decays are usually accompanied by various types of ionizing radiations, consisting of alpha particles (α-ray), electrons (β^−^− and β^+^-ray), and high energy photons (γ-ray). When ionizing radiation interacts with matter, α-ray (helium nuclei flow with weak penetrability and strong ionization property) is easily scattered by electrons and atomic nucleus, while β-ray (electron flow with stronger penetrability and weaker ionization property) is gradually absorbed in cascade interaction with inner shell electrons. Identification of radionuclides is required in many utmost applications such as homeland security, environmental radiation monitoring, and medical diagnostic and therapy^1–4^. However, the identification of radionuclides is often difficult due to rather complex decay process of the parent radioactive element. For example, the decay of radioactive Radon gas generates α-, ß-, and γ- rays with various energy^5^. Identifying the type and energy of ionizing radiation is an urgent and critically important task.

**Fig. 1:**
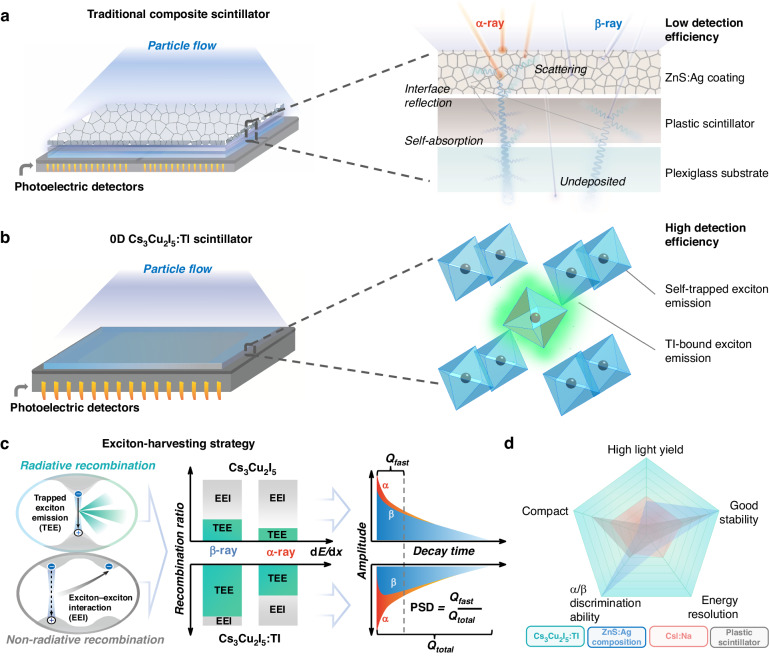
Design concept of α/β discrimination detectors based on high-performance LDH single crystals. **a, b**, Detector structure illustrations of traditional ZnS:Ag/plastic scintillation composites (**a**) and 0D Cs_3_Cu_2_I_5_:Tl single crystals (**b**). **c**, Schematic diagrams of the exciton-harvesting strategy proposed for the enhancement of α- and β-ray discrimination capability in Cs_3_Cu_2_I_5_:Tl single crystals. **d**, Radar chart of the desired properties for α- and β-ray discrimination scintillators. A large radius is desired for a competitive scintillator

The mainstream α- and β-ray discrimination materials are the composite scintillators consisting of ZnS:Ag and polyvinyl toluene (PVT)-based plastics (Fig. 1a)^6^. Thanks to a slow time response of 10 μs under α-ray radiation for ZnS:Ag and a fast time response of 10 ns under β-ray radiation for PVT plastics, the composite detector is able to achieve decent α/β discrimination capability. However, such composite scintillators suffer from inherent low detection efficiency and poor energy identification capability, resulting from the opacity of ZnS:Ag polycrystalline layer, the low detection efficiency of PVT plastics, and multiple interface reflections inside the composite. Afterwards, a series of dual-mode scintillation detection materials, such as CsI:Na single crystals, liquid scintillators, and plastic scintillators, were developed for α/β discrimination applications^7–9^. Nonetheless, they could not be widely utilized in practical scenarios due to the hygroscopic nature of CsI:Na single crystal, and low detection efficiency of liquid and plastic scintillators. Hence, it is highly desirable to develop dual-mode alternatives with high detection efficiency, long-term air stability, and superior discrimination ability^10^.

Recently low-dimensional halides (LDHs), in particular the Cu(I) halides, are attracting great attention as high-atomic-number materials for next-generation scintillators due to their efficient radioluminescence from self-trapped excitons (STE)^11–13^. The advantages of using all-inorganic LDHs for X-ray imaging and γ-ray spectroscopy applications^14–16^, even for α- and β-ray detection, have been proved^17–19^. In LDHs, the charge carriers (and excitons) are found to be strongly localized at inorganic clusters, leading to negligible exciton-defect interaction, large Stokes shift, and high PLQY^20–22^. In principle, the scintillation yield of LDHs should approach their theoretical limits estimated by the Bartram–Lempicki method^23^. However, it was found that most of LDHs has a much lower scintillation yield compared to theoretical limit despite having high PLQY, as seen in Supplementary Fig. S1. It is well known that the exciton-exciton interaction, also known as Auger recombination, is a major quenching channel for halide scintillators under ionizing radiation^24,25^. Thus, the scintillation yield loss in LDHs is most likely associated with the non-radiative quenching caused by the exciton-exciton interaction between adjacent STEs. Meanwhile, since there is a significant difference in the absorption of α- and β-ray for the LDHs, the variance in ionization density, leading to different scintillation decays associated with exciton-exciton interaction recombination ratio, can serve as the basis for α/β discrimination. For instance, under MeV energy radiation, the ionization density of α-ray is about three orders of magnitude higher than that of β-ray. Thus, the potential of LDHs as reliable and high-performance alternatives for charged particle discrimination applications deserves to be explored.

In this work, we propose a new strategy to kill two birds with one stone, namely enhancing scintillation yield and achieving efficient dual-mode α/β discrimination capability in LDHs by exciton-harvesting. We select the 0D Cs_3_Cu_2_I_5_ halides with the superior gamma scintillation yield about 30 000 photons MeV^-1^ among LDHs as the target material. In addition, in previous reports, Tl-doped Cs_3_Cu_2_I_5_ can achieve about a 3-fold increase in light yield, though the mechanism is not clear^14,26,27^. Here, by realizing exciton-harvesting strategy, an efficient Tl-bound exciton emission (trapped exciton emission process, TEE process) is introduced to compete with the host self-trapped exciton emission (also regarded as TEE process), resulting in the reduction of exciton-exciton interaction (EEI) process. Due to the suppression of EEI, the recombination ratio of the TEE process under α- and β-ray can be increased in Cs_3_Cu_2_I_5_:Tl compared to undoped Cs_3_Cu_2_I_5_. Subsequently, the variation in fast decay time associated with the EEI process under α and β ionizing radiation is expected to enhance pulse shape discrimination (PSD) capability in Cs_3_Cu_2_I_5_:Tl. The detector structure based on 0D Cs_3_Cu_2_I_5_ single crystals, and our proposed exciton-harvesting strategy via doping are illustrated in Fig. 1b and c, respectively.

Herein, for the first time, we prove the exciton-harvesting strategy and report the 0D Cs_3_Cu_2_I_5_:Tl single crystals as a highly efficient detection material for α/β discrimination for the first time. Compared with undoped Cs_3_Cu_2_I_5_, Cs_3_Cu_2_I_5_:Tl has a three orders of magnitude enhancement of rejection ratio to 10^-9^, an enhanced α/β PSD FoM factor of 2.64, and also an 86% enhancement in scintillation yield. Compact detectors based on Cs_3_Cu_2_I_5_:Tl single crystals show the capability of identify radioactive ^220^Rn gas for practical environmental monitoring applications. Moreover, Cs_3_Cu_2_I_5_:Tl single crystals also bear some important features that ensure practical applications. First, the Cs_3_Cu_2_I_5_:Tl single crystals can identify nuclides via energy spectra due to its high energy resolution. In contrast, the plastic scintillators are incapable of achieving energy resolution, and ZnS:Ag composite and CsI:Na single crystals scintillators show much worse energy resolution^28,29^. Second, the stability of Cs_3_Cu_2_I_5_:Tl toward humidity and continuous radiation is excellent, compared to hygroscopic CsI:Na single crystals^27,30^. Third, all the contained elements in the host are cheap, abundant, nontoxic, and non-radiative, and the melting point of the compound is low about 390 °C. It guarantees the low production cost and good market potential. The physical and scintillation properties of 0D Cs_3_Cu_2_I_5_:Tl single crystals are comprehensively compared with conventional and some newly developed scintillators in the radar chart (Fig. 1d), exhibiting striking advantages.

## Results and discussion

### Photo-physics of multiple exciton emissions in Cs_3_Cu_2_I_5_:Tl single crystal

**Fig. 2:**
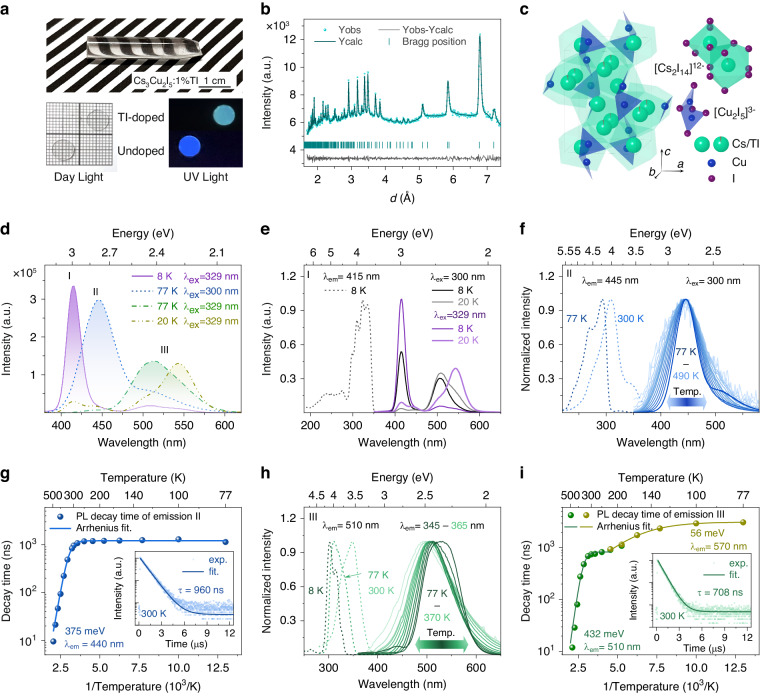
Photo-physics of multiple exciton emissions in Cs_3_Cu_2_I_5_:Tl single crystal. **a**, Cs_3_Cu_2_I_5_:Tl crystal ingot, and the polished slabs under day-light and UV light excitation. **b**, Powder neutron diffraction Rietveld refined results of Cs_3_Cu_2_I_5_:Tl. **c**, Crystal structure configuration of Cs_3_Cu_2_I_5_:Tl. **d**, Three categories of the four emissions of Cs_3_Cu_2_I_5_:Tl. **e, f, h**, Temperature dependence of PL and PLE spectra of emission I (**e**), emission II (**f**) and emission III (**h**). **g, i**, PL decay time as a function of temperature for emission II (**g**) and emission III (**i**), inset shows the decay profiles measured at room temperature

Up to now, the X- and γ-ray detection performance of undoped and Tl-doped Cs_3_Cu_2_I_5_ single crystals were reported based on small size samples, from micrometer to millimeter scale^14,26,27,31^. However, for practical radiation detection applications, it is critically important to grow large volume single crystals with high optical quality by the melting method, such as the Bridgman method. Based on the differential scanning calorimetry, both of Cs_3_Cu_2_I_5_ and Cs_3_Cu_2_I_5_:Tl show severe thermal supercooling and are slightly incongruent (Supplementary Fig. S2), which causes the melt spontaneous nucleation easily into multiple grains and inclusions. Then, temperature-dependent crystal cell parameters were calculated by in-situ synchrotron powder X-ray diffraction Rietveld (Supplementary Fig. S3 and Table. S1-2). A weak thermal expansion anisotropy was characterized. During the cooling process after crystal growth completion, difference in lattice thermal expansion along crystallographic axes will cause the accumulation of thermal stress in the crystal ingots, which results in crystal cracking. To prevent the thermal and constitutional supercooling effect, a long ampoule capillary and large thermal gradient were introduced for the optimization of crystal growth in the Bridgman method. The inclusion- and crack-free 1%Tl-doped Cs_3_Cu_2_I_5_ single crystal ingot with a diameter of 7 mm and length over 30 mm were produced (Fig. 2a). Supplementary Fig. S4 shows the other ingots and Table S3 lists the actual doping concentrations of Cs_3_Cu_2_I_5_:Tl samples. The measured transmittance of Cs_3_Cu_2_I_5_ after cutting and polishing at the maximum emission wavelength (445 nm) is 77.8%, close to the theoretical value of 82.4% (Supplementary Fig. S5-6). For Cs_3_Cu_2_I_5_:Tl, there is even smaller difference between the theoretical (81.9%) and measured values (78.6%) at 505 nm. In addition, the hygroscopic properties of Cs_3_Cu_2_I_5_ and Cs_3_Cu_2_I_5_:Tl were compared with CsI:Na by using the dynamic vapor sorption method. Cs_3_Cu_2_I_5_ and Cs_3_Cu_2_I_5_:Tl show better stability than CsI:Na, shown in Supplementary Fig. S7.

To elucidate the scintillation emission mechanism of Cs_3_Cu_2_I_5_:Tl, the neutron powder diffraction (NPD) technique was used to illustrate the crystal structure configuration, in particular the site occupation of Tl dopants. Figure 2b shows the NPD pattern collected on energy-resolved neutron imaging instrument (ERNI) at room temperature together with the fitting results. By Rietveld refinement on the diffraction data, detailed structural information could be obtained, such as lattice parameters, atomic positions, especially the position of Tl. The neutron diffraction Rietveld refinement fitting plots can be appreciated in Fig. 2b, and the final refined structural parameters are summarized in Table S4. The Rietveld analysis shows slightly decreased lattice parameters, ^*a*^ = ^10.1693(3) Å, *b*^ = ^11.6495(3) Å, *c*^ = ^14.3572(5) Å^, of Cs_3_Cu_2_I_5_:Tl compared with the parent one Cs_3_Cu_2_I_5_^32^. Given that the ionic radii of Tl^+^ is smaller than that of Cs^+^, (1.59 Å of Tl^+^ and 1.74 Å of Cs^+^)^33^, the lattice contraction confirms the successful entrance of Tl into the lattice. To further determine the position of Tl^+^, we tried different refinement models with Tl replacement for Cs, Cu and I. The refinements show that Tl enters the 8*d* site of the lattice i.e. the Cs2 position in Table S5, which is indicated by a slightly improved fitting and decreased χ^2^. Fitting with other models yields either negative *occ* values or positive values with considerable errors.

We continue to study the photo-physics in Tl-doped Cs_3_Cu_2_I_5_. Based on previous results, there are two kinds of emission observed at room temperature: (i) STE emission peaking at 445 nm (2.79 eV) associated with spatially isolated [Cu_2_I_5_]^3-^ clusters, and (ii) Tl-related emission peaking at 510 nm (2.43 eV)^14,27^. The contribution of Tl-related luminescence increased with the Tl-doping concentration, but the emission or excitation wavelengths have no change. A slight acceleration of STE emission and a slight deceleration of Tl-related emission can be observed, which is due to the signal superposition of the two broad emissions (Supplementary Fig. S8)^34^. Here, as shown in Fig. 2d, two other emissions peaking at 415 nm (2.99 eV) and 550 nm (2.25 eV) were observed at low temperature. To understand the origin of the observed emission centers, their temperature dependence of photoluminescence spectra and decay kinetics were systematically studied. All four observed emission bands can be ascribed to three luminescence centers (see Fig. 2d):

*Emission I: Tl*^*+*^ *ions:* Photoluminescence (PL) and excitation (PLE) spectra of emission I are shown in Fig. 2e. Upon monitoring the emission I at 415 nm, two broad excitation bands are observed at 8 K. The weak excitation band range from 200 to 280 nm is associated with the emission II. The main excitation band range from 280 to 350 nm consists of four peaks at 298, 310, 324, and 334 nm, related to the emission I. Under 300 nm and 329 nm excitation at 8 K, the dominant emission I peaks at 415 nm with a full-width at half-maximum (FWHM) of 0.15 eV. The Stokes shift is 0.72 eV. When the measurement temperature rises to 20 K, emission I is significantly reduced, and fully quenched at 40 K (Supplementary Fig. S9). The features of emission I, namely, its PLE and PL spectral position, Stokes shift, and FWHM of the emission peak are very similar to the Tl^+^ emission at 400 nm in CsI host^35^., so that we ascribe the emission I to the Tl^+^ center. However, it is quenched at much lower temperatures (in CsI host the onset of 400 nm band quenching is above 160 K) and its decay, described by two-exponential fit with 0.8 and 1.65 µs decay times at 8 K, see Fig. S10 in Supplementary, is much different from that of 400 nm emission in CsI:Tl which shows about 10 ns and 1 ms decay times in this temperature range. It is most probably due to different local symmetry of Tl^+^ ion in Cs_3_Cu_2_I_5_ host and possible interaction with energy levels of Cu^+^ situated at the bottom edge of the conduction band.

*Emission II: Self-trapped excitons from host lattice:* The normalized PL and PLE spectra of emission II are shown in Fig. 2f. Upon monitoring the emission wavelength of 445 nm, the excitation spectrum at 77 K consists of a main peak at 292 nm and a shoulder peak at 270 nm. There is also an almost invisible weak excitation peak at 330 nm. A redshift of these excitation peaks can be observed when increasing temperature to 300 K. In contrast, with the increase of temperature from 77 to 490 K, the peak position of emission II is nearly the same. The emission II has a broad FWHM of 0.29 eV, and a large Stokes shift of 1.56 eV at 77 K. These are typical features of the STE emission^34^. The formation of STEs needs intense electron-phonon coupling, which is characterized by the Huang-Rhys factor (*S*). In Supplementary Fig. S11, the value of *S* is estimated as 104 by using the Eq. 1. It indicates a strong electron-phonon coupling and supports the STE emission origin^36^. The spectral characteristics of emission II in the Tl-doped Cs_3_Cu_2_I_5_ are identical to STE emission in undoped Cs_3_Cu_2_I_5_ (Supplementary Fig. S12), which is strongly confined in individual [Cu_2_I_5_]^3-^ clusters^32,37^. The measurement of temperature-dependent PL decay of STE emission was performed. The PL decay time as a function of temperature is shown in Fig. 2g. At 8 K, its decay is single exponential with decay time of 2.07 µs, see in Supplementary Fig. S13, and the onset of the thermal quenching occurs around 280 K. The inset in Fig. 2g shows the decay profile at room temperature as an example, in which the single exponential fitting was used. According to the Arrhenius equation (Eq. 2), the exciton binding energy (*E*_a_) is calculated as 340 ± 12 meV. It is consistent with the value of 335 meV reported for the STE in undoped Cs_3_Cu_2_I_5_^38^.

*Emission III: TI-bound excitons:* In addition to Tl^+^ emission and STE emission, there are two other emissions peaking at 510 nm (2.44 eV) and 550 nm (2.26 eV) observed well-resolved at the low temperatures (20-80 K) in Cs_3_Cu_2_I_5_:Tl. These two emissions have very similar characteristics, including the emitted energy, FWHM, Stokes shift, and decay time at low temperatures, except for the thermal stability. They are assigned as emission III. To study the two emissions, the PL-PLE mapping at 77 K was measured and plotted in Supplementary Fig. S14. When monitoring the emission wavelength of 510 nm, the excitation spectrum at 77 K consists of two peaks at 325 and 310 nm. The Stokes shift is 1.36 eV. For the emission wavelength of 550 nm, it is more likely to be excited at 340 nm. The PLE and PL spectra of emission III as a function of temperature are shown in Fig. 2h Similarly with the STEs emission, the excitation peaks of emission III show a redshift when the measurement temperature increases. Since emission III consists of two emission peaks superimposed, we identify the two emissions by bimodal Gaussian fitting of photon energy. Around 220 K, the emission peak at 550 nm is already difficult to identify in PL spectra, see in Supplementary Fig. S15. The *S* and phonon frequency (ℏ*ω*) are evaluated as 62 and 14.6 meV, respectively (Supplementary Fig. S16). At 8 K, the PL decay for the emission at 510 nm is 5 277 ns, and that is 5095 ns for the emission at 550 nm (Supplementary Fig. S17). When the temperature increases to 77 K, the decay times of the two emissions are accelerated differently. The decay profiles at different emission wavelengths (from 490 to 570 nm) were measured when excited at 330 nm (Supplementary Fig. S18). All profiles are single-exponential (It is a sum of two similarly decaying components, but it cannot be identified by the double exponential function because of the similarity of the decay times and overlap of emissions.). The decay times are 2 160 ns monitoring at 490 nm and 3 190 ns monitoring at 570 nm. This indicates that the emission at 510 nm accelerates about 50% faster than that at 550 nm in the 8-77 K temperature range.

**Fig. 3:**
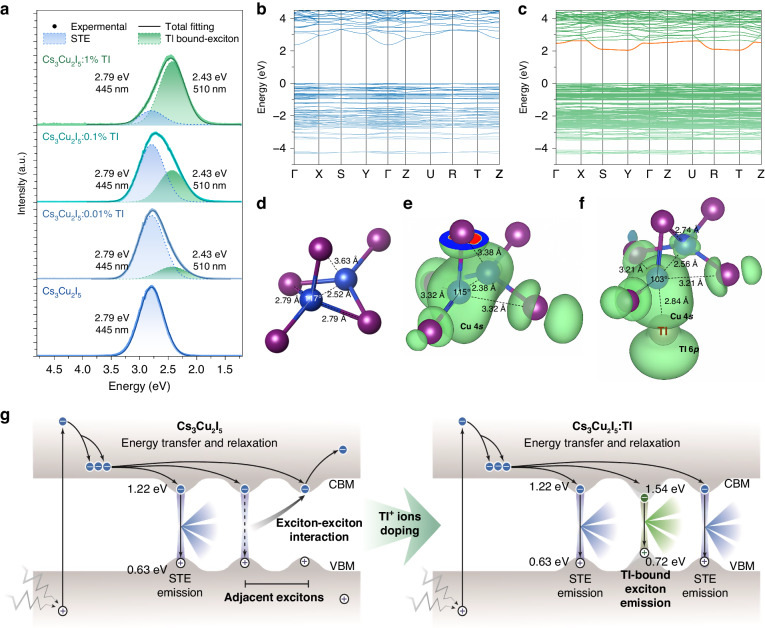
Exciton-harvesting mechanism in Cs_3_Cu_2_I_5_:Tl. **a** Radioluminescence spectra of Cs_3_Cu_2_I_5_:Tl. **b, c** Calculated band structures of Cs_3_Cu_2_I_5_ (**b**) and Cs_3_Cu_2_I_5_:Tl (**c**) by the PBE functional. **d, e, f** Structure and excited electron distribution of [Cu_2_I_5_]^3-^ anion in Cs_3_Cu_2_I_5_:Tl crystal at ground state (**d**), STE state (**e**), and Tl-bound exciton state (**f**) by the HSE calculation. **g** Theoretical single-particle electronic energy levels and scintillation emission mechanisms in Cs_3_Cu_2_I_5_ and Cs_3_Cu_2_I_5_:Tl under ionizing radiation

The temperature dependence of PL decay profiles of emission III were measured when monitoring λ_em_ = 510 nm and λ_em_ = 570 nm from 77 to 490 K. The decay constants of emission III as a function of temperature are plotted in Fig. 3i. Similarly, to PL spectra, the signal associated with 550 nm emission is almost unrecognizable at 220 K. Based on Arrhenius equation (Eq. 2), the exciton binding energy is calculated of 56 meV, indicating a lower exciton thermal stability. For the emission at 510 nm, the value is 432 meV, and it remains stable at room temperature. Its decay time at room temperature is 706 ns. The significant difference between the two temperature ranges (The decay time acceleration of emission at 510 nm is faster in 8-77 K and slower in 77-220 K compared to that of emission at 550 nm) suggests that the two emission centers are not completely independent. The features of large Stokes shift, broad emission peak, high Huang-Rhys factor, and long decay time suggest the nature of trapped exciton emission for emission III. However, it is clear that the decay time evolution trend as a function of the temperature of emission III is different from the host STE emission. It slowly decreases from 77 to 350 K, and then rapidly decreases at higher temperature. This situation closely resembles the temperature dependence decay kinetics of the ns^2^ ions (such as Tl^+^, Pd^2+^, and Bi^3+^) bound exciton emission^39–42^. At the lowest temperatures (8-20 K), it clearly show thermally stimulated bidirectional transitions between both the adiabatic potential excited state (APES) minima (Fig. S17) which closely resembles the situation in CsI:Tl^35^. Thus, the emission III can be ascribed to the two configurations of exciton trapped around an adjacent Tl ion (Tl-bound exciton), similar to the Tl-bound exciton emission observed in CsI:Tl^40,43^. Furthermore, based on the NPD refinement results mentioned above, the Cs site with nine-fold coordination is more favorable for Tl doping. The thermally stimulated transitions between the 510 nm and 550 nm occur at much lower temperatures compared to CsI:Tl, similarly to the 410 nm emission of Tl^+^, which points to significantly lower energy barriers between their APES minima.

## Exciton-harvesting mechanism in Cs_3_Cu_2_I_5_:Tl

To get deep insight on the mechanism in Cs_3_Cu_2_I_5_ and Tl-doped Cs_3_Cu_2_I_5_, the types of non-radiative recombination are analysed. The emission features of trapped excitons under radiation are plotted in Fig. 3a. Decomposing by Gaussian function, the radioluminescence spectra of Tl-doped Cs_3_Cu_2_I_5_ crystals are composed by the STE and Tl-bound exciton emissions, and their ratio is negatively correlated with Tl concentration. In other words, all the scintillation luminescence is from the recombination of excitons. It makes the exciton-exciton reaction a key point in the study of the scintillation mechanism of Cs_3_Cu_2_I_5_:Tl crystals. In fact, the interaction between the adjacent excitons has been observed in many inorganic scintillators^25,44,45^, particularly in halides. Here, the changes of electron, hole, and trapped excitons of Cs_3_Cu_2_I_5_ after Tl doping should be clarified.

In order to get the character of the band edge and the shape of the band structure, the first-principle electronic structure calculations were performed by the Perdew-Burke-Ernzerhof (PBE) functional at first. The decomposed partial density of states shows that the valance band maximum (VBM) is consisting of Cu 3*d* and I 5*p* states, while the conduction band minimum (CBM) is consisting of Cu 4 *s* orbitals. Due to the isolation of [Cu_2_I_5_]^3-^, the band structure of Cs_3_Cu_2_I_5_ is relatively flat as shown in Fig. 3b, which favors the formation of localized exciton^46,47^. For Tl-doped Cs_3_Cu_2_I_5_, only the 8*d* site is considered because its formation energy is lower by 0.18 eV than that of 4*c* site, consistent with the NPD analysis. The calculated band structure of Tl-doped Cs_3_Cu_2_I_5_ shows that Tl_Cs_ introduces 6*p* orbitals below the conduction band (Fig. 3c). However, the PBE method will underestimate the calculated band gap (*E*_g_) value^47^.

So as to describe the photo-physical properties more accurately, the following electronic structure and excited states calculations were performed using more advanced Heyd-Scuseria-Ernzerhof (HSE) method. The calculated emission energy of STE is 2.76 eV, which is consistent well with our experimental result of 2.79 eV. Its binding energy (*E*_a_ = *E*_STE_*-E*_perfect cell_*-E*_g_) is 0.48 eV, where *E*_STE_ and *E*_perfect cell_ are the total energies of the STE and the perfect cell employed. The calculated emission energy of Tl-bound exciton is 2.09 eV, which is also close to the experimental value of 2.43 eV. The structure of [Cu_2_I_5_]^3-^ anion in the ground state is shown in Fig. 3d. After the excitation of the electrons to the conduction bands, lattice distortion occurs within the [Cu_2_I_5_]^3-^ anion. The calculations of structural relaxation show that the two Cu ions move closer with a bond length of 2.38 Å, compared with the perfect case of 2.52 Å. Correspondingly, two Cu-I bonds are broken with a long distance of 3.32 Å, while they are of 2.79 Å in ground state. The single-particle level of electron is 1.22 eV lower than CBM and that of the hole is 0.63 eV higher than VBM for STE. The excited electrons are mainly *s* orbital electrons and surround two nearby Cu ions (shown in Fig. 3e), while the holes are in Cu *d* orbital. For the Tl-bound exciton, the Cu and Tl ions move towards each other with distance of 2.84 Å, and the two Cu-I bonds are broken with distance up to 3.21 Å. Here, the interaction between the two Cu ions is even weaker than that in STE state, with a distance of 2.56 Å. The excited electrons occupy the *p* orbital of Tl and *s* orbital of Cu (shown in Fig. 3f), which are hybridized due to their close energies. The single-particle levels of electron and hole in Tl-bound exciton are 0.32 and 0.09 eV deeper than those of STE, respectively, which indicates that the formal excitons are more localized and more stable because of the weaker exciton interaction originated from the overlap of wavefunction. Therefore, the Tl-bound exciton is more difficult to migrate with less probability to encounter nonradiate center^47^.

Based on the above analysis, the proposed scintillation mechanisms in undoped and Tl-doped Cs_3_Cu_2_I_5_ excited by ionizing radiation are put forward and illustrated in Fig. 3g. For 0D Cs_3_Cu_2_I_5_, after being irradiated with high-energy particles/rays, an avalanche of secondary electrons is produced, thermalized, and coupled with surrounding crystal lattice to form STEs due to the soft lattice nature of LDHs, and emits photons finally^32,37^. However, a large part of excitons will non-radiatively decay through the exciton-exciton interaction^45,48^. It may explain an overlooked and non-intuitive phenomenon that 0D and 1D halides have a high PLQY (sometimes near-unity), but the measured scintillation yield is far less than their theoretical values (Fig. S1). By exciton-harvesting via Tl doping into Cs_3_Cu_2_I_5_ lattice, the formation of Tl-bound excitons can efficiently eliminate the exciton-exciton interaction processes, and then enhance the radiative recombination probability. Moreover, based on thermoluminescence (TL) profiles of undoped and Tl-doped Cs_3_Cu_2_I_5_, as shown in Fig. S19 in Supplementary, Tl doping does not induce new TL peaks throughout the entire measurement temperature, indicating no introduction of new traps^49^. Considering significant enhancement of scintillation yield by isovalent doping of Tl, the considerable increase of TL intensity in Tl doped sample cannot be ascribed to the increase of trap density, but rather to the reinforcement of radiative emission probability at recombination centers via exciton-harvesting.

## α- and β-ray detection and discrimination performance

**Fig. 4:**
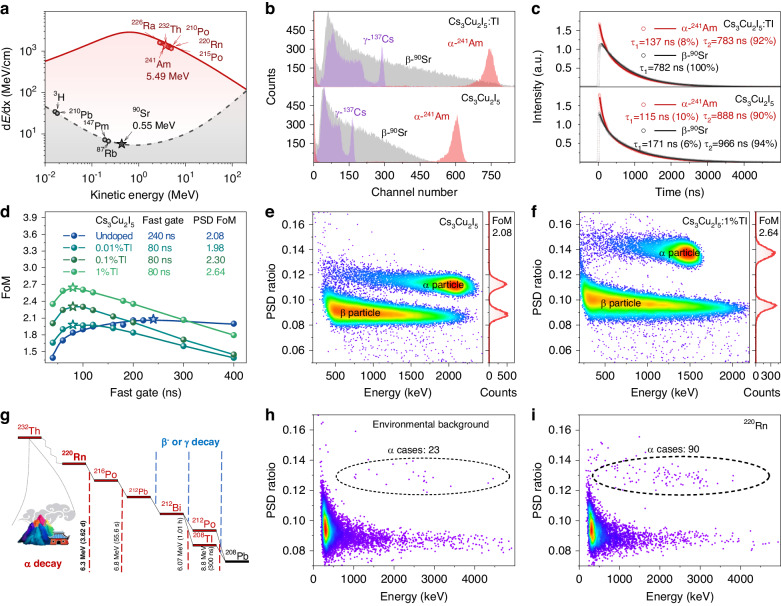
α- and β-ray detection and discrimination performance. **a** Ionization density of Cs_3_Cu_2_I_5_ as a function of energy under α- and β-ray excitation. **b** Energy spectra under the excitation of ^137^Cs, ^241^Am and ^90^Sr sources. **c**, Averaged scintillation pulse waveforms under the excitation of ^241^Am and ^90^Sr sources. **d** FoM values as a function of fast gate window. **e, f** PSD scatter plots and spectra of Cs_3_Cu_2_I_5_ and Cs_3_Cu_2_I_5_:1%Tl under the excitation of ^241^Am and ^90^Sr sources. **g**, Source and brief decay scheme of ^220^Rn in the natural environment. **h, i**, PSD scatter plots of Cs_3_Cu_2_I_5_:Tl under (**h**) environmental background and (**i**) ^220^Rn excitation

Generally, the non-radiative exciton-exciton quenching is supported by the appearance of fast components in the scintillation decay. In addition, the suppression of exciton-exciton interaction through efficient harvesting should be more pronounced for high-ionization density excitation compared to low-ionization density excitation. In other words, in principle, Tl doping can enhance the distinction between scintillation pulses generated by high and low ionization density irradiation. The exciton-harvesting mechanism by Tl doping increases the radiative recombination fraction and strengthens the difference of pulse shape under radiation of different ionizing densities. Thus, it makes Cs_3_Cu_2_I_5_:Tl as well as other low-dimensional halide scintillation crystals potentially an efficient multimode detection material. We conducted a comprehensive evaluation of the detection and discrimination performance of the undoped and Tl-doped Cs_3_Cu_2_I_5_ single crystals for the α- and β-ray detection. Specifically, we analyzed the energy spectra, scintillation decay, and α/β PSD analysis using ^241^Am (α source, mainly emitting α particles with an energy of 5.49 MeV) and ^90^Sr (β source, continuously distributed energy with a maximum energy of 2.28 MeV). For Cs_3_Cu_2_I_5_, ionization density as a function of incident energy, namely d*E*/d*x*, under α- and β-ray excitation is plotted in Fig. 4a. For radiation with an energy around 1 MeV, the ionization density under α-ray excitation is about three orders of magnitude higher than that under β-ray excitation. High-density ionization will lead to high probability of exciton-exciton interaction, resulting in a fast scintillation decay component appearance. This effect serves as the basis for α/β PSD in undoped Cs_3_Cu_2_I_5_.

Figure 4b shows the pulse height spectra of undoped and Tl-doped Cs_3_Cu_2_I_5_ single crystals under excitation by ^137^Cs, ^241^Am and ^90^Sr. The full-energy peak is well-resolved under α-ray excitation for both the undoped and Tl-doped Cs_3_Cu_2_I_5_, and a continuously distributed energy spectrum is observed under β-ray excitation. The absolute light yields were determined by the single photoelectron technique via Eq. 3. The value for α-ray of undoped Cs_3_Cu_2_I_5_ is 14,000 photons MeV^-1^ and reaching 26,000 photons MeV^-1^ for 1%Tl-doped Cs_3_Cu_2_I_5_. To further confirm the light yield calibration, the light yield under 662 keV γ-ray excitation is estimated as 31,000 photons MeV^-1^ for undoped Cs_3_Cu_2_I_5_ to 83 000 photons MeV^-1^ for Cs_3_Cu_2_I_5_:1%Tl. The enhancements of scintillation yield (86% for α-ray and 168% for γ-ray as shown in Supplementary Fig. S20) should be mainly attributed to the suppression of exciton-exciton quenching, especially for the low-ionization density excitation. Additionally, pulse height spectra of Cs_3_Cu_2_I_5_ and Cs_3_Cu_2_I_5_:Tl under ^137^Cs, ^133^Ba, and ^152^Eu γ-ray excitation were acquired in Supplementary Fig. S21. Cs_3_Cu_2_I_5_:Tl exhibits smaller light yield non-proportionality than that of Cs_3_Cu_2_I_5_. Obtained by R6233-100 PMT with higher quantum efficiency in Supplementary Fig. S22, the α-ray energy resolution of Cs_3_Cu_2_I_5_ is 10.2% at 5.49 MeV and it is 10.5% of 1%Tl-doped Cs_3_Cu_2_I_5_. The α-ray photopeak shows an asymmetric shape, mainly because the alpha decay of ^241^Am includes two branches of 5 486 keV (85.2%) and 5 442 keV (12.8%); in addition, the alpha particles also lose energy when travelling through the air to the sample. In contrast, as shown in Supplementary Fig. S23, the spectrum of commercial ZnS:Ag/plastic composite scintillators under ^241^Am excitation shows a broad and unresolved full-energy peak due to the heterogeneous structure and the poor energy resolution of ZnS:Ag. The comparison of continuum spectra under ^90^Sr source excitation indicates a much higher β-ray detection efficiency in Cs_3_Cu_2_I_5_:Tl single crystals than that of composite scintillators.

The averaged scintillation decays under the α- and β-ray excitation are shown in Fig. 4c. Normalizing to the same integral area means the same equivalent electron energy. The decay of undoped crystals under α- and β-ray excitation can be fitted well by a double-exponential function. Excitation under ^241^Am source, with a high ionization density, results in a 10% intensity of fast component decay with 115 ns, whereas excitation by the ^90^Sr source leads to a 6% intensity of fast component decay with 171 ns. Increasing the Tl doping concentration results in a lower relative intensity and deceleration of the fast component under α- and β-excitations (Supplementary Fig. S24). Notably, for the sample with the highest Tl concentration (1 at%), a 137 ns fast component decay is observed under ^241^Am excitation, whereas it is undetectable under ^90^Sr excitation. The Tl doping concentration increase accelerates the slow component associated with the trapped exciton emissions. Meanwhile, the fast decay component decelerates and its proportion is reduced.

The manipulation of α/β PSD capability in Cs_3_Cu_2_I_5_ single crystals doped with Tl was based on the variation of fast and slow components during α- and β-ray excitation. The FoM value represents the discrimination capability, for which Figure of Merit (FoM) = 1 was usually considered as a minimum required limit^50^. The α/β PSD ratios are plotted onto the Y-axis in a 1D projection distribution that calculates the FoM value by Gaussian fitting and Eq. 4. Undoped Cs_3_Cu_2_I_5_ single crystals demonstrated the α/β PSD capability with FoM of 2.08. More importantly, a considerable enhancement of FoM value from 1.98 to 2.64 is achieved in 1%Tl-doped Cs_3_Cu_2_I_5_ single crystals via optimizing the integration time windows and Tl doping concentration (Fig. 4d). In Fig. 4e, f and Supplementary Fig. S25, each point on the 2D scatter plot represents a particle event, where α and β rays are distinct. The rejection ratio, here referring to the probability of β ray being misidentified as an α ray, is also used to measure the identification ability. Theoretically, when the FoM value is raised from 2.08 to 2.64, the rejection ratio is raised from 10^-6^ to 10^-9^, meaning that only 1 in 100 million β rays will be misinterpreted as an α ray^51^.

By measured radioactive Radon gas, the α-ray detection sensitivity of Cs_3_Cu_2_I_5_:Tl was evaluated. Radon and its decay daughters are natural radioactive sources, and widely exist in the natural environment, especially in soil, building materials, and precipitation (Fig. 4g)^5,52,53^. Radiation damage can be caused in the human respiratory system as a result of α-rays produced during the decay process of ^220^Rn, along with β and γ radiations, which is one of the primary causes of lung cancer^54^. A preliminary radon detection test was performed using the device shown in Supplementary Fig. S26. After loading ^220^Rn into the gas wash bottle, 90 more α events were detected during the same duration of the test, as shown in Fig. 4h and Fig. 4i. Although the experimental conditions were insufficient to quantify the concentration of ^220^Rn, the identification of α-rays confirms the potential of Rn detectors based on Cs_3_Cu_2_I_5_:Tl single crystals.

## Conclusions

To summarize, we have shown the great potential of 0D Cs_3_Cu_2_I_5_ single crystals serving as charged particle discrimination scintillators, and also demonstrate the significant improvements in scintillation yield and α/β ray discrimination capability by exciton-harvesting strategy. Tl-doping introduces the Tl-bound excitons, leading to significant suppression of exciton-exciton quenching. Benefiting from that, the Cs_3_Cu_2_I_5_:Tl can achieve a higher scintillation yield of 26 000 photons MeV^-1^ under α-ray excitation, a more remarkable α/β PSD FoM of 2.64, and a superior rejection ratio of 10^-9^ (Table S6). As being compared, the scintillation yield of Cs_3_Cu_2_I_5_ is 14 000 photons MeV^-1^ under α-ray excitation and the α/β PSD FoM is 2.08. We also demonstrate the capability of identifying radioactive ^220^Rn by Cs_3_Cu_2_I_5_:Tl single crystals, suggesting its promising applications in the environmental monitoring field. With its many other advantages, such as non-hygroscopic nature, manufacturing scalability, low use cost and high performance, Cs_3_Cu_2_I_5_:Tl could be regarded as promising scintillation material for next-generation of α/β detection and discrimination applications. More importantly, this work opens up a new horizon for designing high-performance scintillators based on LDHs with efficient exciton emissions.

## Materials and methods

### Single crystal preparation

High-quality Cs_3_Cu_2_I_5_:Tl single crystals were grown by the vertical Bridgman method. Anhydrous CsI, CuI, and TlI of 99.999% purity in beaded form were used as starting raw materials. The Tl doping concentration is 0, 0.01, 0.1, and 1 at%, assuming that Tl substitutes at Cs site. The formular is (Cs_1-x_Tl_x_)_3_Cu_2_I_5_ (x = 0, 0.0001, 0.001, and 0.01). Process details can be found in Supplementary Information. Single crystal samples were processed by slow wire cutting and polished by micron Al_2_O_3_ powder.

### Crystal structure analysis

NPD measurements were carried out on the ERNI at China Spallation Neutron Source (CSNS), China. The NPD data were analyzed using the Rietveld package FULLPROF SUITE^55^.

### Photo-physics study

The temperature-dependent PL spectra from 77 to 500 K were measured by an Edinburgh Instruments FLS 980 spectrometer excited by a Xe lamp. The PLE and PL spectra, and PL decays from 8 to 80 K were measured by using a custom-made 5000 M Horiba Jobin Yvon spectrofluorometer equipped with a photon counting detector TBX-04 (IBH Scotland). A deuterium steady stay-lamp (Heraeus Gmbh) was used as the excitation source for spectral acquisition. A pulsed nano-LED was used as the excitation source for PL decays acquisition.

The Huang-Rhys factor (*S*) is obtained by nonlinear fitting the temperature dependence Fwhm values with the equation:

1 $$\begin{array}{c}{Fwhm}=2.36\sqrt{S}{{{\hslash }}\omega }_{{\rm{phonons}}}\sqrt{\coth \frac{{{{\hslash }}\omega }_{{\rm{phonons}}}}{2{k}_{{\rm{B}}}T}}{\rm{\#}}\end{array}$$

where *ℏω*_phonons_ is the phonon frequency.

The relationship between decay time (*τ*) and temperature (*T*) is described by the following equation:

2 $$\begin{array}{c}\dfrac{1}{\tau }=\dfrac{1}{{\tau }_{0}}+\omega \exp \left(\dfrac{{E}_{{\rm{a}}}}{-{k}_{{\rm{B}}}T}\right){\rm{\#}}\end{array}$$

where *τ*_0_ is the decay time measured at the lowest temperature, *ω* is the frequency factor, *k*_*b*_ is the Boltzmann constant, and *E*_a_ is the activation energy of thermal quenching.

## Theoretical calculation

The calculation of band composition is based on PBE functional. The electronic structure and photophysical properties calculations are based on the HSE hybrid functional^56^ implemented in the Vienna ab initio simulation package (VASP)^57^. The mixing parameter for the nonlocal Hartree-Fock exchange was set to be 0.27 for the band gap calculation. The cut off energy for the plane wave basis was set at 400 eV and the atomic positions were fully relaxed until the residual forces are less than 0.02 eV Å^-1^. The 40-atom cell with experimental lattices (*a* = 10.42 Å, *b* = 11.84 Å, and *c* = 14.59 Å) is used for our calculations and the Brillouin zone (BZ) integration is sampled by *Γ* point mesh.

## Particle detection and discrimination

The X-ray excitation radioluminescence spectra were measured using an X-ray tube (W target, 50 kV, 500 μA) as the excitation source and an Ocean Optical CCD spectrometer.

The crystal samples and ZnS:Ag/plastic scintillator were coupled to the photomultiplier tubes (PMT). A calibrated Hamamatsu R2059 PMT was used to obtain the light yield by using the single photopeak method and a Hamamatsu R6233-100 was for α- and β-rays detection and discrimination measurements^58^. The light yield is calculated by the following equation:

3 $$\begin{array}{c}{Light\; yield}=\frac{{{Ch}}_{{\rm{sample}}}}{{{Ch}}_{{\rm{s}}.{\rm{g}}.}\cdot {EWQE}\cdot {E}_{{\rm{R}}}}=\frac{{{Ch}}_{{\rm{sample}}}\cdot \int \left[I\left(\lambda \right)\cdot {QE}\left(\lambda \right)\right]}{{{Ch}}_{{\rm{s}}.{\rm{g}}.}\cdot \int I\left(\lambda \right)\cdot {E}_{{\rm{R}}}}{\rm{\#}}\end{array}$$

where *Ch*_sample_ and *Ch*_s.g._ are the channel numbers of the full-energy peak and the single photoelectron peak. *EWQE* is the emission-weighted quantum efficiency. *I(λ)* and *QE(λ)* are radioluminescence intensity and the R2059 PMT detection efficiency as a function of emission wavelength, respectively. *E*_R_ is the energy of radiation rays. The radioactive sources used in the experiments were ^137^Cs (γ ray, 0.662 MeV, 1.17 × 10^6 ^Bq), ^241^Am → ^237^Np + ^4^He (α ray, 5.49 MeV, 3.46 × 10^4 ^Bq) and ^90^Sr → ^90^Y + e^-^ (β ray, maximum of 2.28 MeV, 3.02 × 10^8 ^Bq). The samples were cladded with the optical silicone grease and covered with Teflon tape to improve scintillation light collection efficiency. During the α ray test, a small hole was made in the Teflon tape to avoid the absorption of α particles. The operation voltage of R2059 PMT was 1700 V and that of R6233-100 PMT was 1400 V. The anode signals were directly recorded using a CAEN DT5751 digitizer (1 GHz/10-bit).

The original waveform data were then processed offline using the ROOT package software. The original waveform data for each particle was integrated and filled into a histogram to obtain the energy spectrum. To obtain a representative average waveform of α or β rays, every 100 waveforms were averaged. The charge comparison method, which uses the ratio of the charge integrated into the fast gate and total gate (*PSD ratio* = *Q*_f_/*Q*_t_), was used for PSD. The ROOT package software was used to offline process the PSD parameter of each event from waveform data of α or β rays, which were then filled into the 2D histogram. By projecting the 2D scatter plot onto the Y-axis, a 1D projection distribution of α and β rays could be obtained. The figure-of-merit (FoM) was used to quantify the ability to PSD and calculated as:

4 $$\begin{array}{c}{FoM}=\frac{D}{{{Fwmh}}_{{\rm{\alpha }}}+{{Fwmh}}_{{\rm{\beta }}}}{\rm{\#}}\end{array}$$

where *Fwmh*_α_ and *Fwmh*_β_ are the full widths at half-maximum values of the α and β peaks in the 1D histogram of the PSD parameter, and *D* is the distance between the two peak positions. They are obtained by fitting the two peaks with Gaussian functions.

### Supplementary information

Supplementary Information for Exciton-Harvesting Enabled Efficient Charged Particle Detection in Zero-Dimensional Halides
