# Supplementary material for: Exciton-harvesting enabled efficient charged particle detection in zero-dimensional halides
Source: Light Sci Appl. 2024 Aug 14;13:190. doi: 10.1038/s41377-024-01532-z (PMC11322634; doi:10.1038/s41377-024-01532-z)
Supplement: Supplementary file 1 — Supplementary Information for Exciton-Harvesting Enabled Efficient Charged Particle Detection in Zero-Dimensional Halides [file 41377_2024_1532_MOESM1_ESM.docx]

**Supplementary** **Information for**

Exciton-Harvesting Enabled Efficient Charged Particle Detection in Zero-Dimensional Halides

Qian Wang^1†^, Chenger Wang^2,3†^, Hongliang Shi^4†^, Jie Chen^2,5^, Junye Yang^2,5^, Alena Beitlerova^6^, Romana Kucerkova^6^, Zhengyang Zhou^1^, Yunyun Li^1^, Martin Nikl^6^, Xilei Sun^2,^*, Xiaoping OuYang^7,^*, and Yuntao Wu^1,^*

*^1^Shanghai Institute of Ceramics, Chinese Academy of Sciences, Shanghai, 201899, China.*

*^2^Institute of High Energy Physics, Chinese Academy of Sciences, Beijing, 100049, China.*

*^3^National Engineering Research Center for Rare Earth, Grirem Advanced Materials Co., Ltd. and General Research Institute for Nonferrous Metals; Beijing, 100088, China.*

*^4^Department of Physics, Beihang University; Beijing, 100191, China.*

*^5^Spallation Neutron Source Science Center, Dongguan 523803, China*

*^6^Department of Optical Materials, Institute of Physics of the Czech Academy of Sciences; Prague, 16200, Czech Republic.*

*^7^Northwest Institute of Nuclear Technology; Xi’an, 710024, China.*

†These authors contributed equally to this work.

*Correspondence author. Email: sunxl@ihep.ac.cn (X. Sun), oyxp2003@aliyun.com (X. OuYang), ytwu@mail.sic.ac.cn (Y. Wu)

**Difference between theoretical and measured light yield**

The light yields and photoluminescence quantum yields (PLQYs) of low-dimensional copper-based halide scintillators are listed by statistics. The theoretical light yields are estimated according the reported band gap values by Bartram–Lempicki model^1^:

$$\begin{aligned} LR=\frac{S*Q}{\beta*E_{g}}\#1 \end{aligned}$$

where *S* and *Q* are the transport/transfer efficiency of the e–h pair energy to the emission centers and the internal quantum efficiency in emission centers, respectively. Here, the values are set as 1. *β* is 1.5–1.8 for ionic halide compounds and the approximate mean is used as 1.65.

**
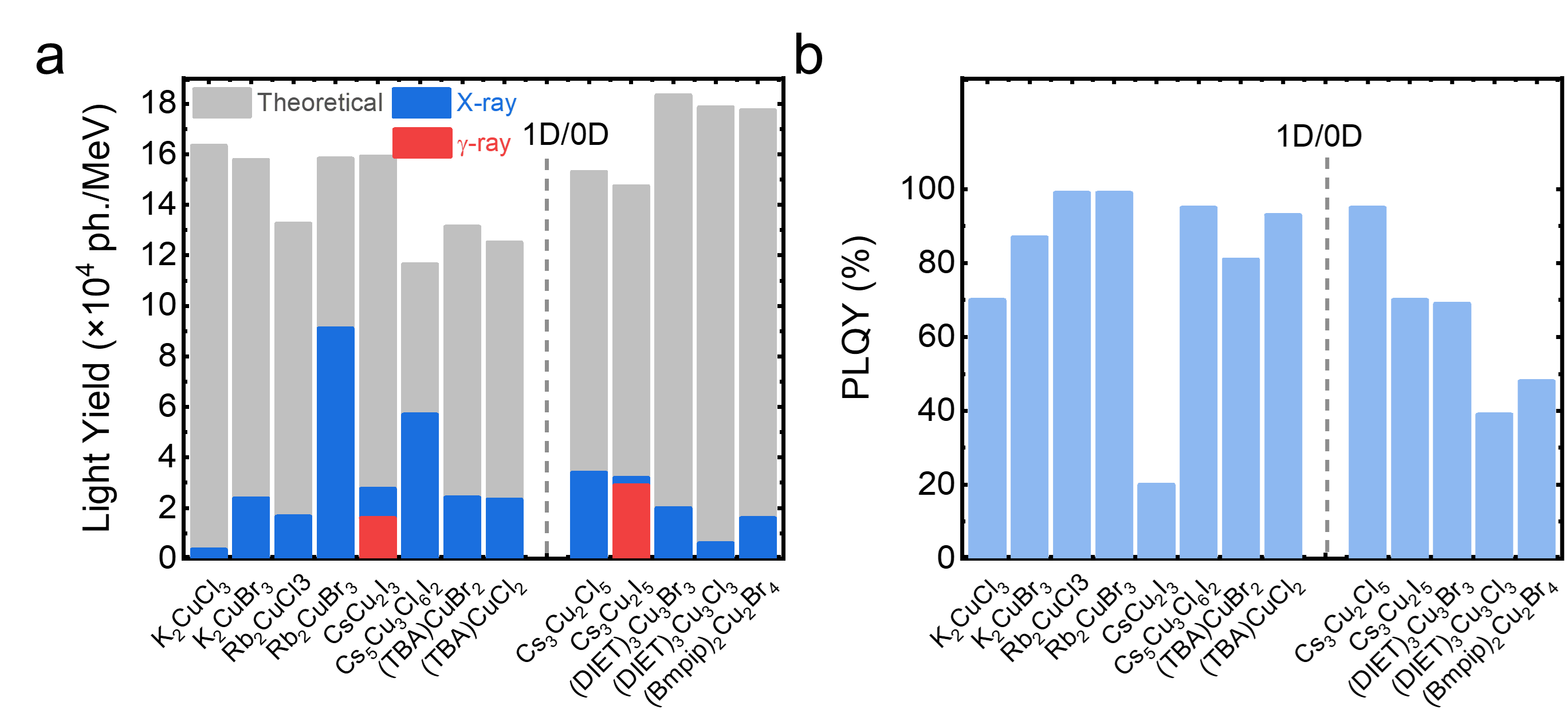
**

Figure S1. a, b, Light yields (a) and PLQYs (b) of low-dimensional copper-based halide scintillators.^2, 3, 4, 5, 6, 7, 8, 9, 10, 11, 12, 13, 14^

**Crystallographic property**

The differential scanning calorimetry (DSC) measurement was conducted by using a Netzsch STA449F3 instrument. About 50 mg single crystal was loaded in an Al_2_O_3_ crucible, and heated to 450 °C at a rate of 5 °C/min under ultra-high purity argon atmosphere.

**Figure S2.** DSC profiles of Cs_3_Cu_2_I_5_ and Cs_3_Cu_2_I_5_:Tl in Ar atmosphere.

There is no additional thermal effect induced by Tl doping. The Cs_3_Cu_2_I_5_ shows a pair of exothermic and endothermic peaks at 396 °C and 329 °C corresponding to the melting point (*T*_m_) and crystallization point (*T*_c_). Compared with undoped Cs_3_Cu_2_I_5_, the *T*_m_ and *T*_c_ decrease slightly in Cs_3_Cu_2_I_5_:Tl. It is worthwhile to note that for both the undoped and Tl-doped Cs_3_Cu_2_I_5_, the thermal supercooling, which causes spontaneous nucleation in the melt into multiple grains, is severe due to a large temperature difference between *T*_m_ and *T*_c_, e.g. 67 °C for Cs_3_Cu_2_I_5_ and 70 °C for Cs_3_Cu_2_I_5_:Tl. Moreover, there is an additional exothermic peak at the lower temperature side of the main melting peak. It can be interpreted as the second phase precipitated due to the incongruent melting of Cs_3_Cu_2_I_5_.^15^ The CsI phase precipitated first (389 °C, L_0.60CsI+0.40CuI_→CsI+L) during the temperature decrease. When the ratio of CsI in the melt is reduced to 0.59, the crystallization process is transformed and the Cs_3_Cu_2_I_5_ main phase is precipitated (329 °C, L_0.59CsI+0.41CuI_ → Cs_3_Cu_2_I_5_).

Temperature-dependent in-situ synchrotron powder X-ray diffraction (PXRD) measurements were conducted by using a PANalytical X'Pert diffractometer using Cu Kα radiation. The crystal lattice parameters at different temperatures were calculated by Le bail fitting of PXRD patterns through Jana2006.


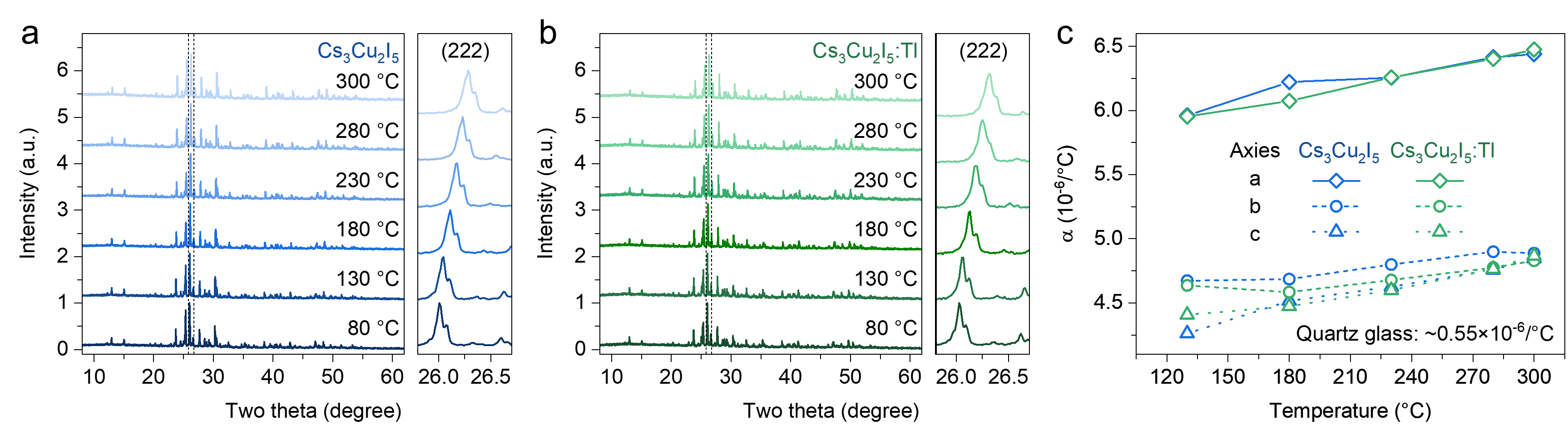


**Figure S3.** **a, b,** Temperature-dependent in-situ PXRD patterns of Cs_3_Cu_2_I_5_ (a) and Cs_3_Cu_2_I_5_:Tl (b). **c,** Crystallographic evolution of thermal expansion coefficients as a function of temperature.

The in-situ PXRD measurements from room temperature to 300 °C were conducted. The XRD patterns as a function of temperature are shown in Fig. S3a and Fig. S3b. The XRD peaks shift to a lower angle due to the thermal expansion effect, for example for (222) crystallographic plane, the angle shifts from 26.283° to 26.008° for Cs_3_Cu_2_I_5_, and 26.317° to 26.028° for Cs_3_Cu_2_I_5_:Tl. The lattice parameters were calculated at different temperatures via the Le bail fitting, and listed in Table S3-4.

The evolution of thermal lattice expansion coefficients along crystallographic axes as a function of temperature is plotted in Fig. S3c. The thermal expansion behavior in Cs_3_Cu_2_I_5_ and Cs_3_Cu_2_I_5_:Tl is similar. The thermal expansion coefficient along *b* and *c* axis is of 4.3 × 10^-6^ and 4.8 × 10^-6^ °C^-1^, respectively, which is about 25% smaller than that along *a* axis ranging from 6.0 × 10^-6^ °C^-1^ to 6.5 × 10^-6^ °C^-1^. It indicates rather weak thermal expansion anisotropy in both Cs_3_Cu_2_I_5_ and Cs_3_Cu_2_I_5_:Tl, comparing with the LaBr_3_:Ce that is apt to crack due to a large difference in thermal expansion coefficient along crystallographic axis (28.1 × 10^-6^ °C^-1^ along *a* axis and 7.5 × 10^-6^ °C^-1^ along *c* axis).^16^

Table S1. Lattice parameters of Cs_3_Cu_2_I_5_ at different temperatures.

| Temperature (°C) | *a* (Å) | *b* (Å) | *c* (Å) | *Pnma*  *α* = *β*= *γ* = 90° |
| --- | --- | --- | --- | --- |
| 25 | 10.17475 | 11.65392 | 14.36231 |  |
| 80 | 10.20575 | 11.68294 | 14.39435 |  |
| 130 | 10.23617 | 11.71024 | 14.42502 |  |
| 180 | 10.26926 | 11.73768 | 14.45934 |  |
| 230 | 10.30154 | 11.76704 | 14.49425 |  |
| 280 | 10.33668 | 11.79741 | 14.53136 |  |
| 300 | 10.35036 | 11.80856 | 14.54783 |  |

Table S2. Lattice parameters of Cs_3_Cu_2_I_5_:Tl at different temperatures.

| Temperature (°C) | *a* (Å) | *b* (Å) | *c* (Å) | *Pnma*  *α* = *β*= *γ* = 90° |
| --- | --- | --- | --- | --- |
| 80 | 10.19706 | 11.67873 | 14.38901 |  |
| 130 | 10.22742 | 11.7058 | 14.42072 |  |
| 180 | 10.25900 | 11.73227 | 14.45337 |  |
| 230 | 10.29278 | 11.76069 | 14.48828 |  |
| 280 | 10.32767 | 11.79028 | 14.52614 |  |
| 300 | 10.34233 | 11.80279 | 14.54313 |  |

Based on the above analysis results, we stress the importance of ampoule design and crystal growth process. The quartz ampoule with a straight capillary was used to prevent the thermal supercooling effect. A large temperature gradient of 30-40 °C and a slow growth rate of 0.3-0.5 mm h^-1^ were adopted to avoid the constitutional supercooling effect.

The raw materials were weighed according to the formula, and loaded into a quartz ampoule with 7 mm inner diameter in an Argon-filled glovebox. The loaded raw materials were heated to 120 °C under vacuum of 10^-4^ Pa to remove any trace moisture prior to sealing with an oxy-hydrogen torch. The crystals were grown from a randomly oriented seed formed in a quartz ampoule with a capillary at the front. Moreover, to avoid the adverse reactions between the crystal and the surface of quartz ampoule, the ampoule was coated with a durable pyrolytic carbon before use.

The crystal growth was conducted in a home-made multi-ampoule vertical Bridgman furnace. The loaded ampoules were first heated to above the melting point of Cs_3_Cu_2_I_5_ for several hours to fully mix and homogenize the melt. After optimization, the translation rate and temperature gradient are found to be 0.4 mm h^-1^ and 20~25 °C cm^-1^, respectively. After growth completion, the furnace was cooled to room temperature at 5 °C h^-1^.

The ingots of Cs_3_Cu_2_I_5_ (a), Cs_3_Cu_2_I_5_:0.01%Tl (b), and Cs_3_Cu_2_I_5_:0.1%Tl (c) were shown in Fig. S4. The ingots were cut in the same position as possible to obtain the samples. High quality wafers were gotten after grinding and polishing.


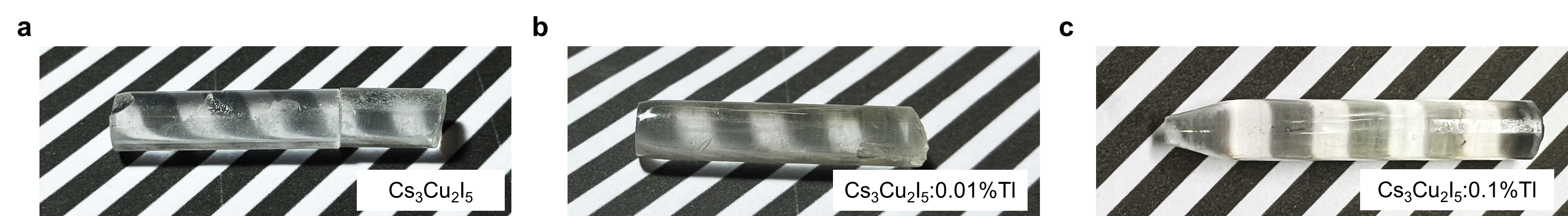


**Figure S4. a, b, c,** Ingots of Cs_3_Cu_2_I_5_ (a), Cs_3_Cu_2_I_5_:0.01%Tl (b), and Cs_3_Cu_2_I_5_:0.1%Tl (c).

The actual Tl concentrations were measured by an Agilent 5100 inductively coupled plasma-optical emission spectrometry (ICP-OES) , as shown in Table S3. It can be found that the actual concentrations of 0.1% and 1% Tl doped samples are about 1/5 of the doping concentration, which can be attributed to the segregation effect of Tl. The actual concentration of 0.01% Tl sample is about 1/2 of the 0.1% Tl doped sample. Nonetheless, the 0.01% Tl doped sample still could be used for Tl-concentration dependent comparison.

Table S3. Actual Tl concentrations of Cs_3_Cu_2_I_5_:Tl samples.

| Sample | Mass fraction  (wt.%) | Average  (wt.%) | Atomic fraction  (at.%) |
| --- | --- | --- | --- |
| Cs_3_Cu_2_I_5_:0.01%Tl-1 | 0.0095 | 0.009(7) | 0.011% |
| Cs_3_Cu_2_I_5_:0.01%Tl-2 | 0.0098 |  |  |
| Cs_3_Cu_2_I_5_:0.01%Tl-3 | 0.0096 |  |  |
| Cs_3_Cu_2_I_5_:0.1%Tl-1 | 0.0165 | 0.016(7) | 0.0195% |
| Cs_3_Cu_2_I_5_:0.1%Tl-2 | 0.0166 |  |  |
| Cs_3_Cu_2_I_5_:0.1%Tl-3 | 0.0168 |  |  |
| Cs_3_Cu_2_I_5_:1%Tl-1 | 0.183 | 0.181(7) | 0.212% |
| Cs_3_Cu_2_I_5_:1%Tl-2 | 0.181 |  |  |
| Cs_3_Cu_2_I_5_:1%Tl-3 | 0.181 |  |  |

**Evaluation of crystal quality**

The optical transmission spectra from 200 to 800 nm were recorded by the PerkinElmer-Lambda 950 ultraviolet-visible spectrophotometer. Refractive index and extinction coefficient spectra were obtained using a sopra GES-SE ellipsometer.


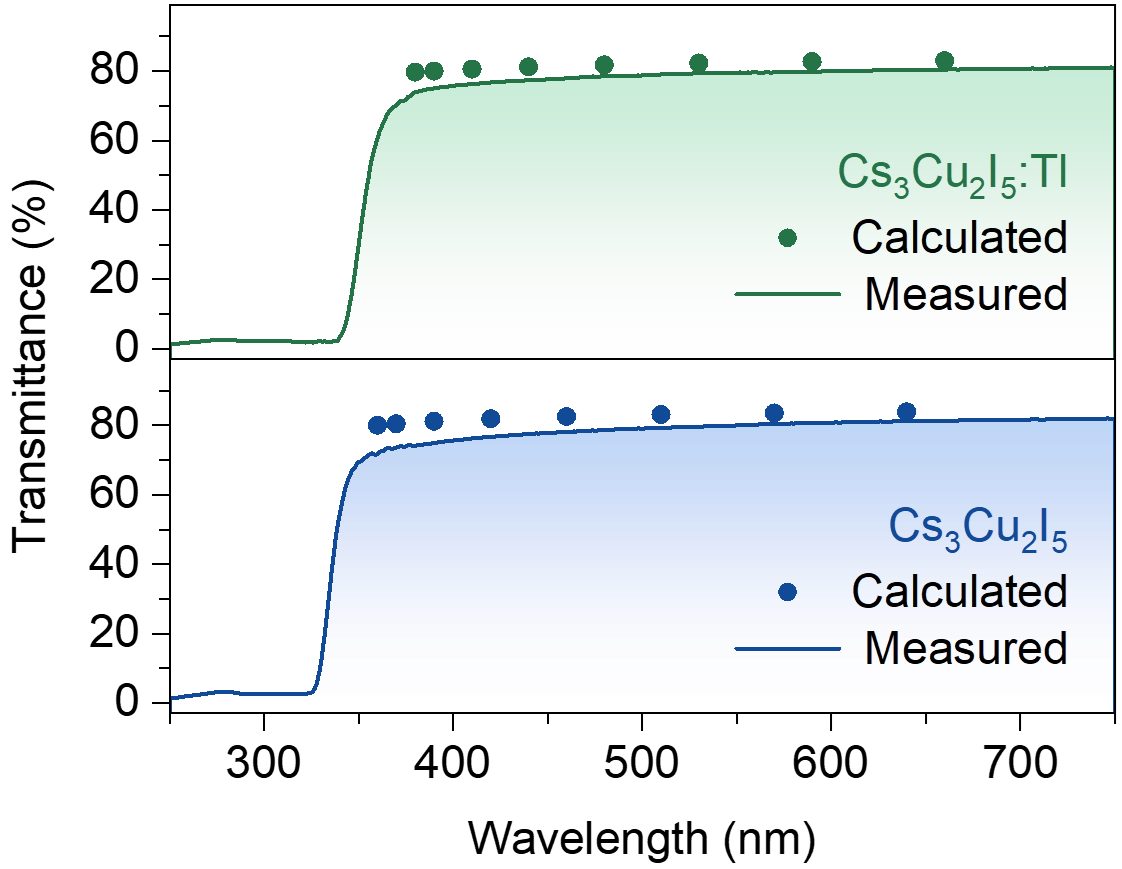


**Figure S5.** Optical transmission spectra and the theoretical values of crystal slabs.

The theoretical transmittance (*T*_s_) at different wavelengths was calculated by using the following equation^17^:

$$\begin{aligned} T_{s}=\frac{2n_{\mathrm{crystal}}n_{\mathrm{air}}}{n_{\mathrm{crystal}}^{2}+n_{\mathrm{air}}^{2}}\#2 \end{aligned}$$

where *n*_crystal_ and *n*_air_ are the refractive index of crystal and air as a function of temperature, respectively. The value of *n*_air_ is 1.0027. The refractive index at the maximum emission wavelength is 1.90 (445 nm) of Cs_3_Cu_2_I_5_ and 1.92 (505 nm) of Cs_3_Cu_2_I_5_:Tl.


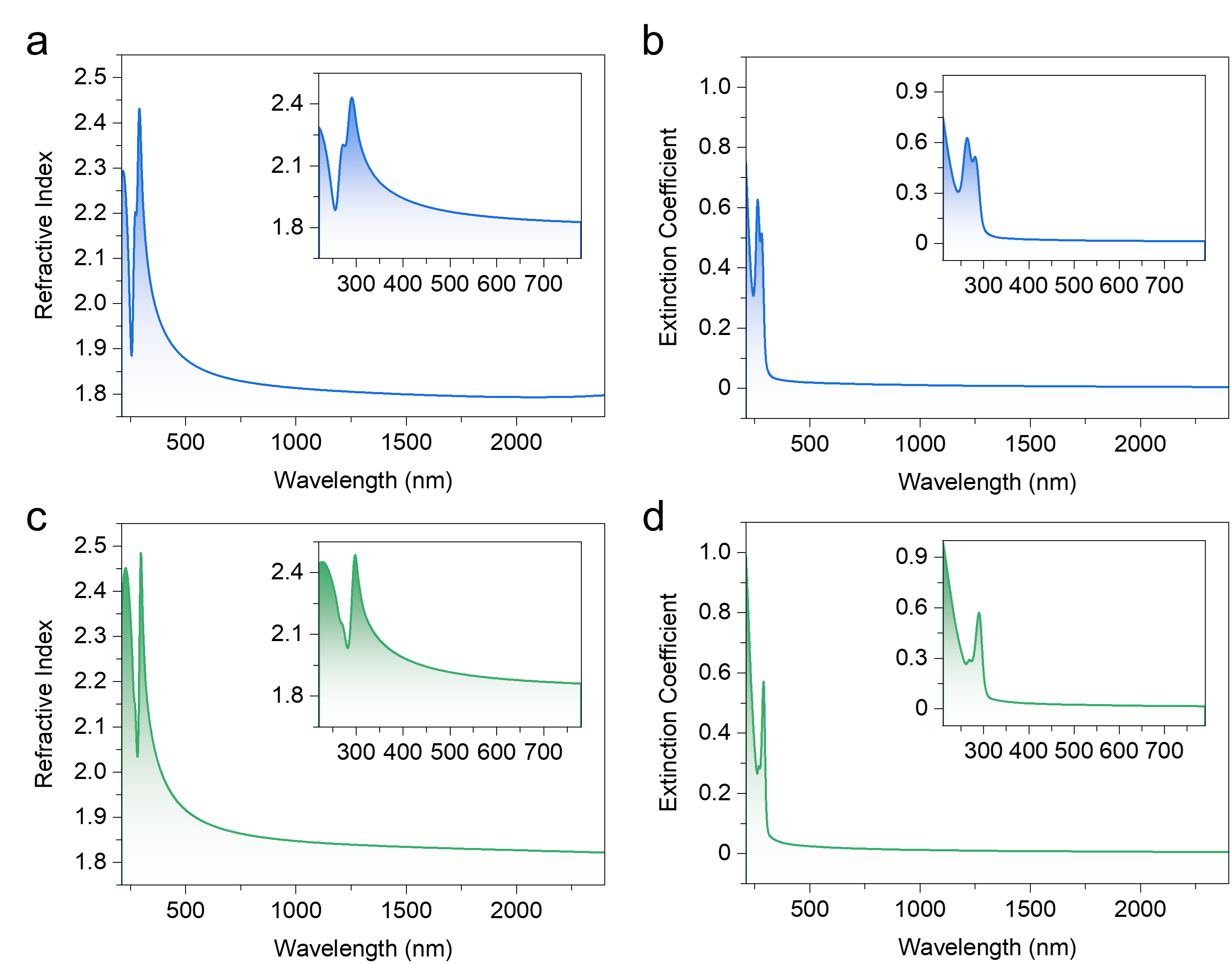


Figure S6. a, b, Refractive indexes (a) and extinction coefficients (b) of Cs_3_Cu_2_I_5_ single crystal. c, d, Refractive indexes (c) and extinction coefficients (d) of Cs_3_Cu_2_I_5_:Tl single crystal.

The hygroscopicity of Cs_3_Cu_2_I_5_, Cs_3_Cu_2_I_5_:Tl and CsI:Na was compared by gravimetric analysis. A Dynamic Vapor Sorption (DVS) instrument SMS DVS Intrinsic Plus was used to continuously measure the mass gain curves under identical conditions: 25 °C, 70% relative humidity, and 2 h. The samples are individual crystal grains of about 50 mg.


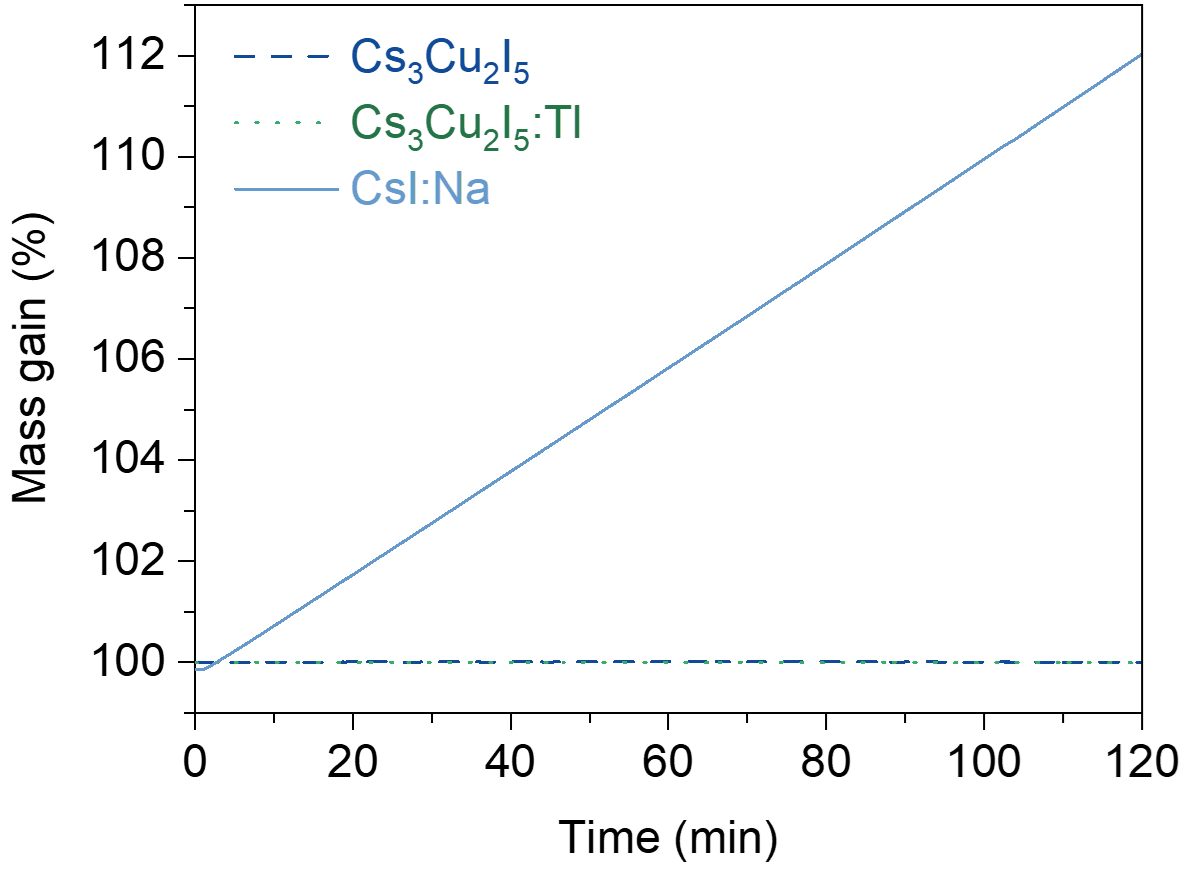


**Figure S7.** Mass gain curves of Cs_3_Cu_2_I_5_, Cs_3_Cu_2_I_5_:Tl and CsI:Na.

**Rietveld** **refinement by NPD**

Table S4. Crystallographic parameters of Cs_3_Cu_2_I_5_:Tl obtained by NPD data at room temperature.

| Sample | Cs_3_Cu_2_I_5_:Tl |
| --- | --- |
| Temperature | 298 K |
| Crystal system | Orthorhombic |
| Space group | *Pnma* (No. 62) |
| Unit cell parameters | *a* = 10.1693(3) Å, α = 90°  *b* = 11.6495(3) Å, β = 90°  *c* = 14.3572(5) Å, γ = 90° |
| *R*_wp_ | 11.5% |
| *R*_p_ | 8.69% |
| *χ^2^* | 1.30 |

Table S5. Atomic positions of Cs_3_Cu_2_I_5_:Tl from NRD Rietveld refinement.

| Atoms | Sites | *x* | *y* | *z* | *occ* |
| --- | --- | --- | --- | --- | --- |
| Cs1 | 4*c* | 0.59156 | 0.25000 | 0.55287 | 1.00 |
| Cs2 | 8*d* | 0.05377 | 0.98806 | 0.67951 | 0.98(1) |
| Tl | 8*d* | 0.05377 | 0.98806 | 0.67951 | 0.02(1) |
| Cu1 | 4*c* | 0.24017 | 0.25000 | 0.37443 | 1.00 |
| Cu2 | 4*c* | 0.21845 | 0.25000 | 0.55227 | 1.00 |
| I1 | 8*d* | 0.69170 | 0.55827 | 0.55047 | 1.00 |
| I2 | 4*c* | 0.15729 | 0.25000 | 0.20011 | 1.00 |
| I3 | 4*c* | 0.97013 | 0.25000 | 0.50940 | 1.00 |
| I4 | 4*c* | 0.29978 | 0.25000 | 0.71805 | 1.00 |

**Photoluminescence**

The PL emission and excitation contour mappings were measured with a Horiba Jobin Yvon Fluorolog-3 spectrofluorometer. With the increase of Tl concentration, the relative intensity of Tl-related emission to STE emission increases clearly. For the Cs_3_Cu_2_I_5_:1%Tl sample, it is almost entirely dominated by Tl-related emission. The variations in luminescent center spectral shapes observed during contour mapping are attributed to the overlapping of spectra and coloring deviation.


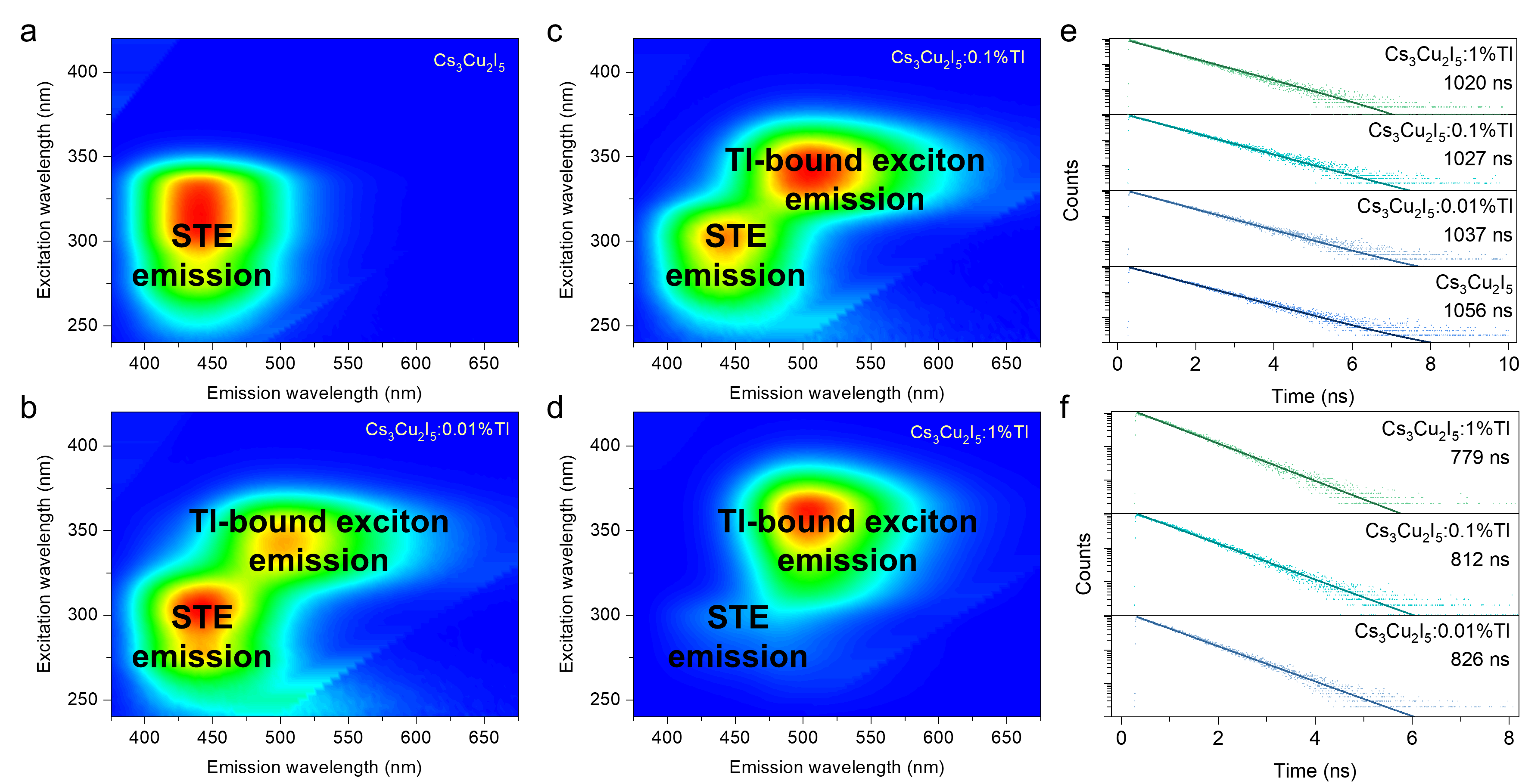


Figure S8. a, b, c, d, PL and PLE spectra mapping of Cs_3_Cu_2_I_5_ (a), Cs_3_Cu_2_I_5_:0.01%Tl (b), Cs_3_Cu_2_I_5_:0.1%Tl (c), and Cs_3_Cu_2_I_5_:1%Tl (d). e, f, Doping concentration dependent PL decay profiles of STE emission (e, λ_ex_ = 280 nm, λ_em_ = 440 nm) and Tl-related emission (f, λ_ex_ = 330 nm, λ_em_ = 530 nm) at room temperature.

**
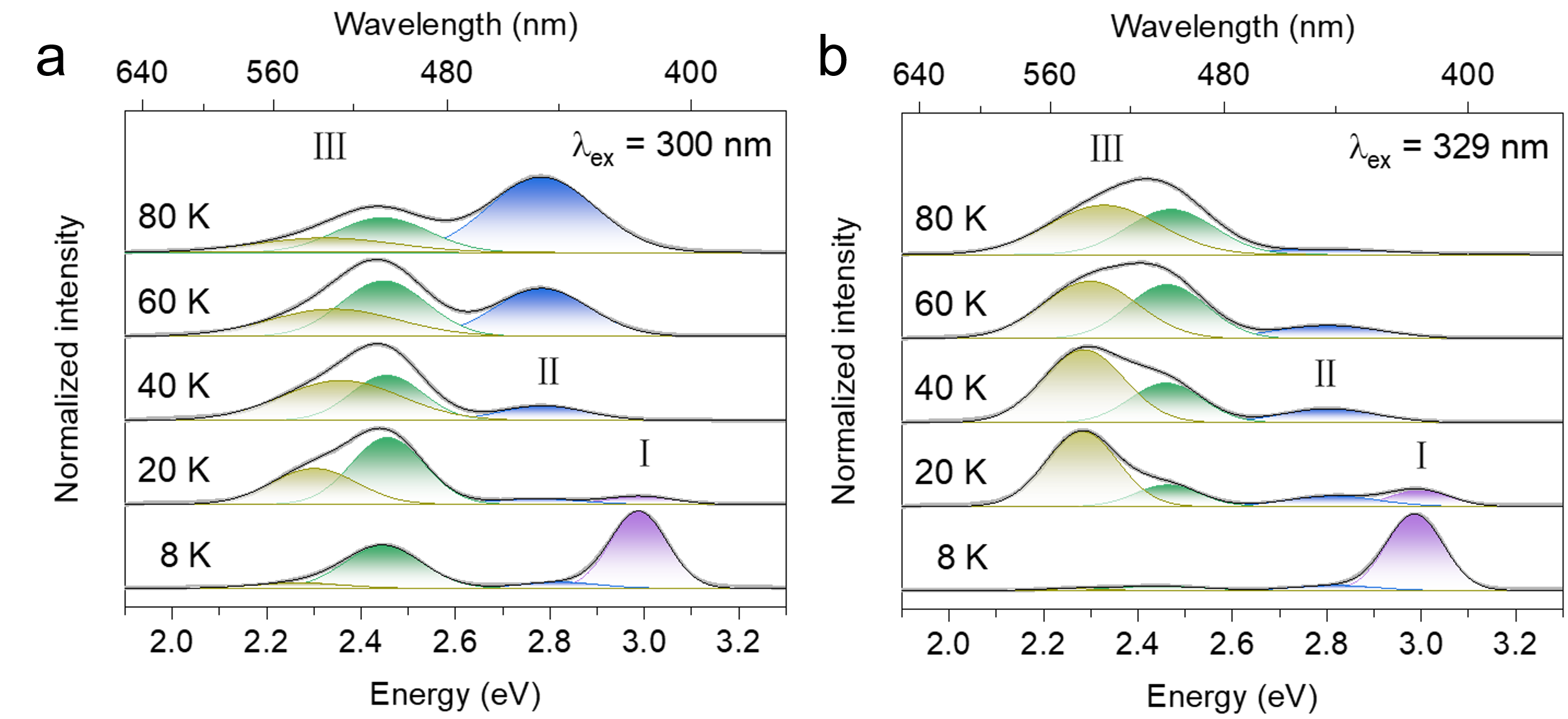
**

Figure S9. a, b, Temperature-dependent PL spectra of Cs_3_Cu_2_I_5_:Tl single crystal under 300 nm (a) and 329 nm (b) excitation below 80 K.

^
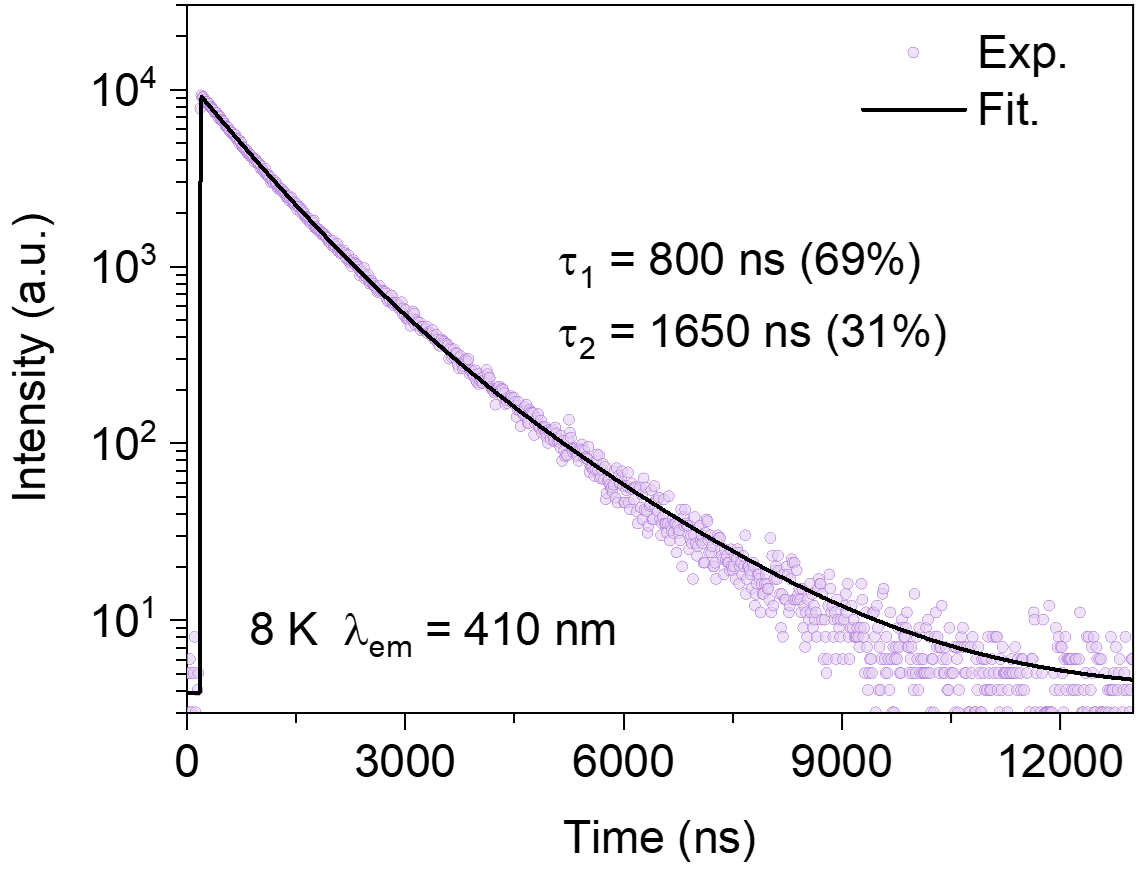
^

Figure S10. PL decay profile of 410 nm (2.99 eV) emission in Cs_3_Cu_2_I_5_:Tl single crystal at 8 K.


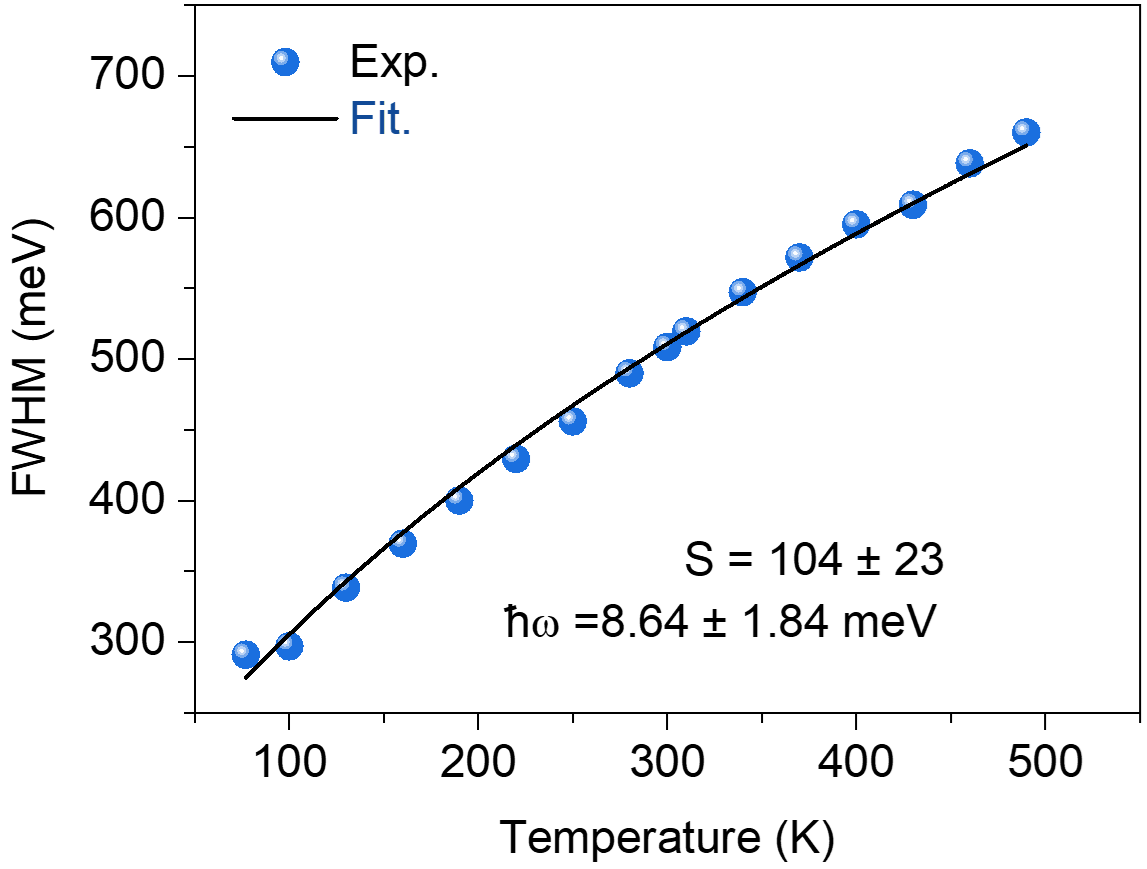


Figure S11. Full width at half maximum (*FWHM*) of STE emission (445 nm) as a function of temperature. The fitted Huang-Rhys factor (*S*) and phonon frequency (*ℏω*_phonons_) are listed in the figure.

**
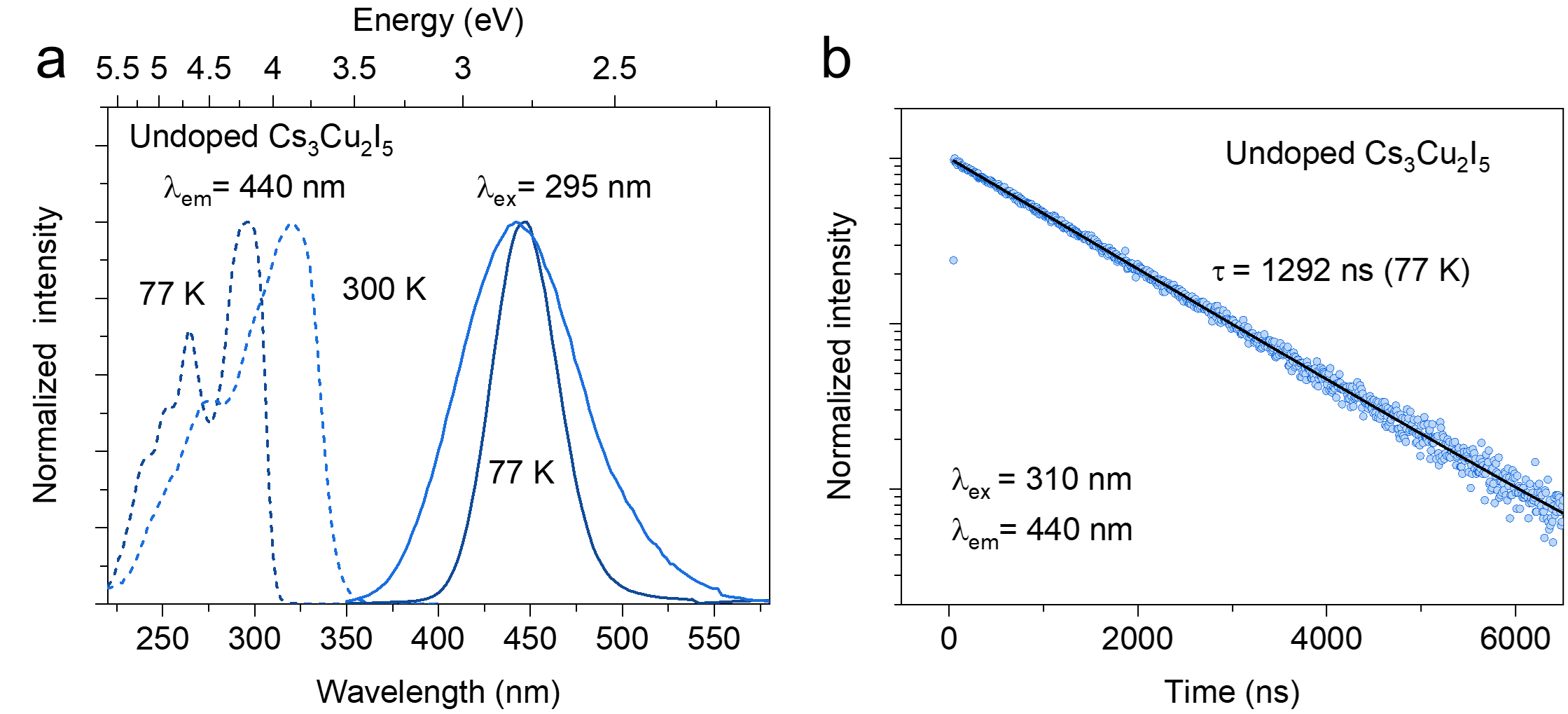
**

Figure S12. a, PL and PLE spectra of Cs_3_Cu_2_I_5_ at 77 and 300 K. b, PL decay time profile of Cs_3_Cu_2_I_5_ at 77 K


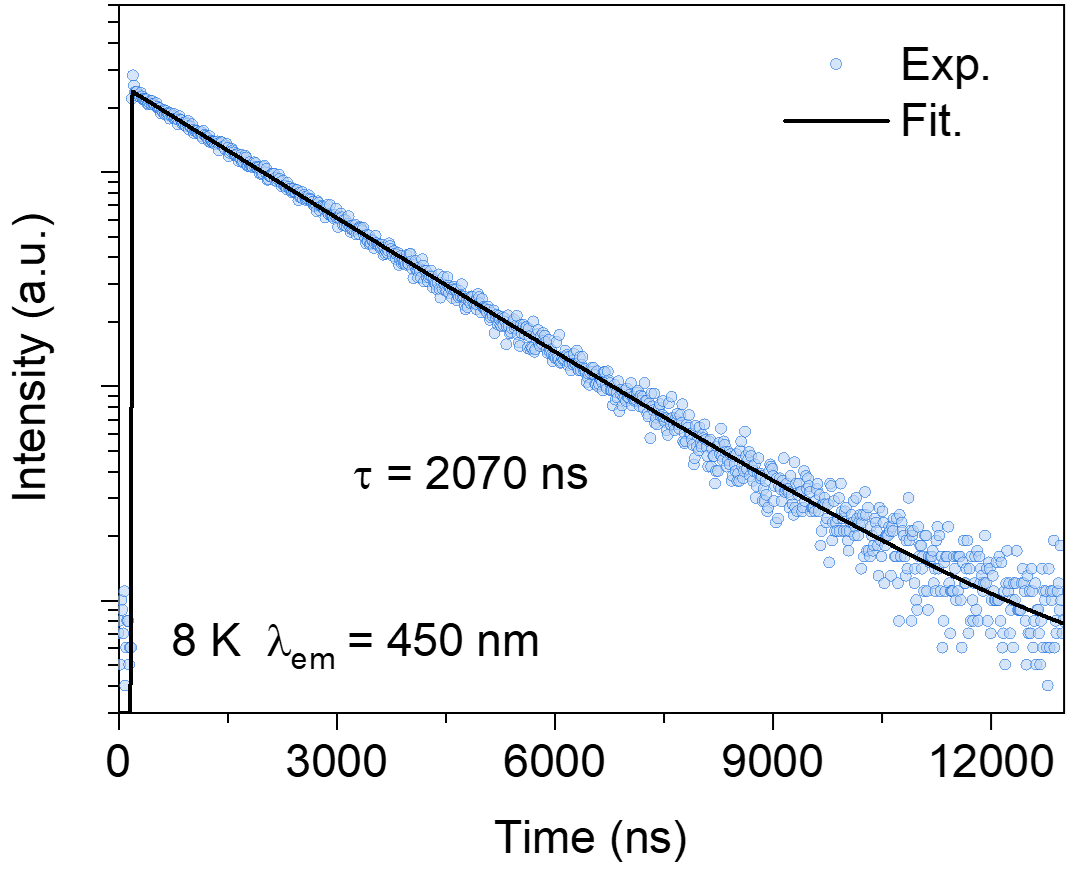


Figure S13. PL decay profile of 445 nm (2.79 eV) emission in Cs_3_Cu_2_I_5_:Tl single crystal at 8 K.


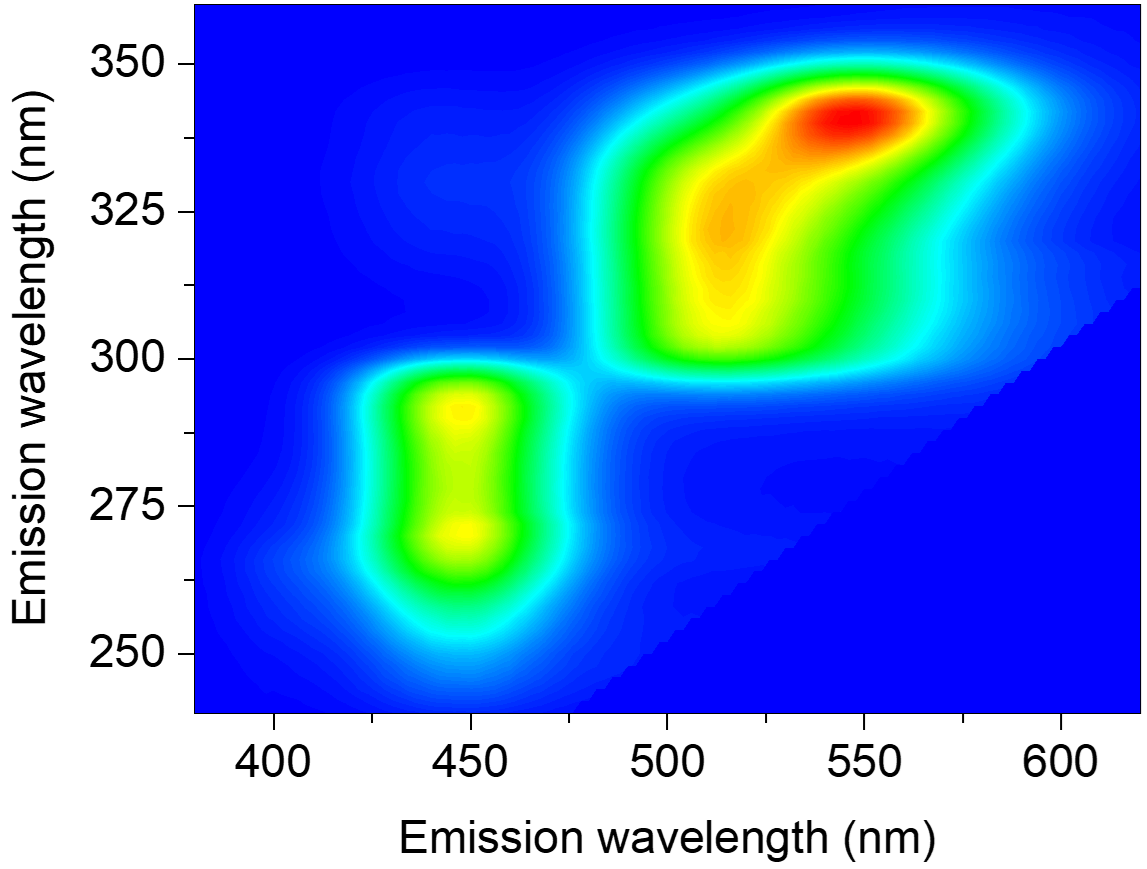


Figure S14. PL-PLE mapping of Cs_3_Cu_2_I_5_:Tl single crystal at 77 K.


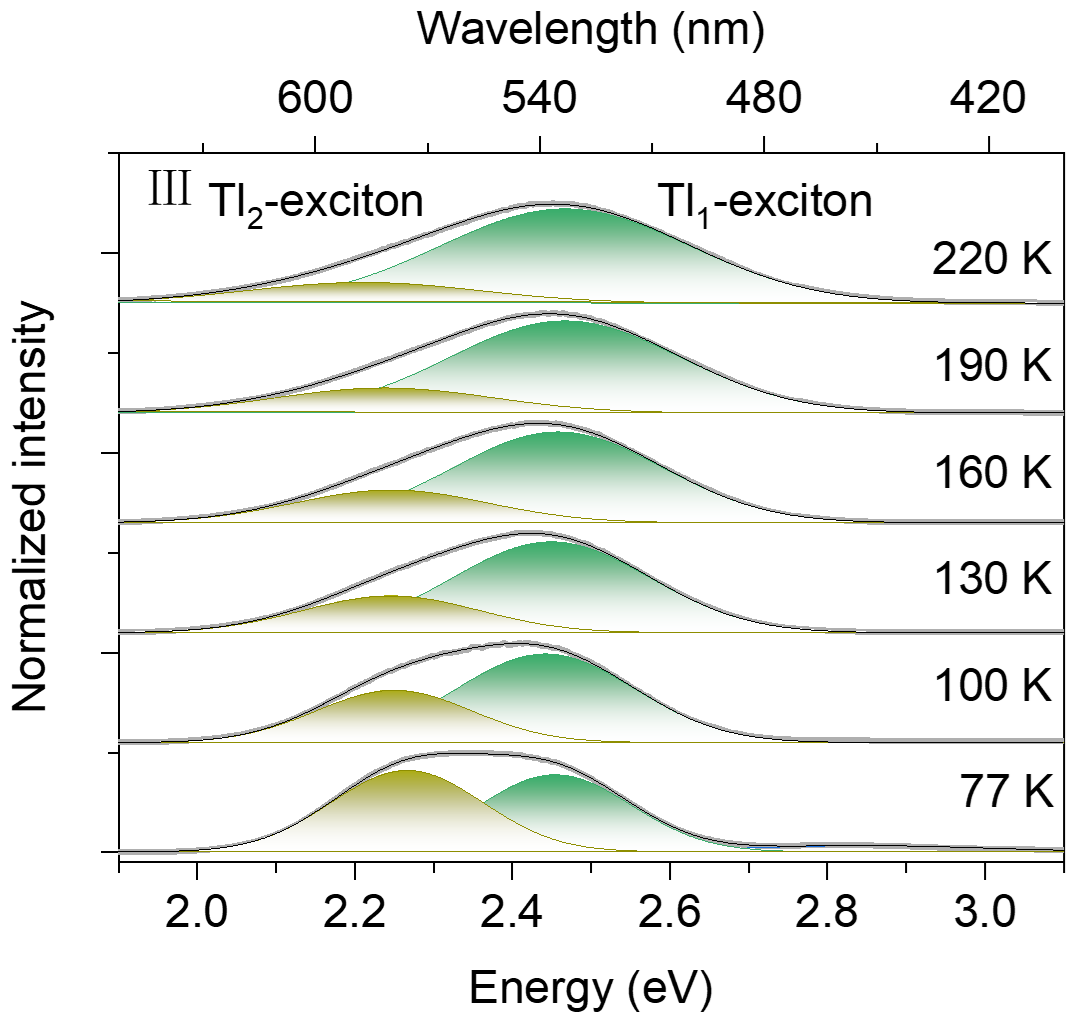


Figure S15. Two-peak gaussian fitting of Tl-bound exciton emission spectra from 77 to 220 K.


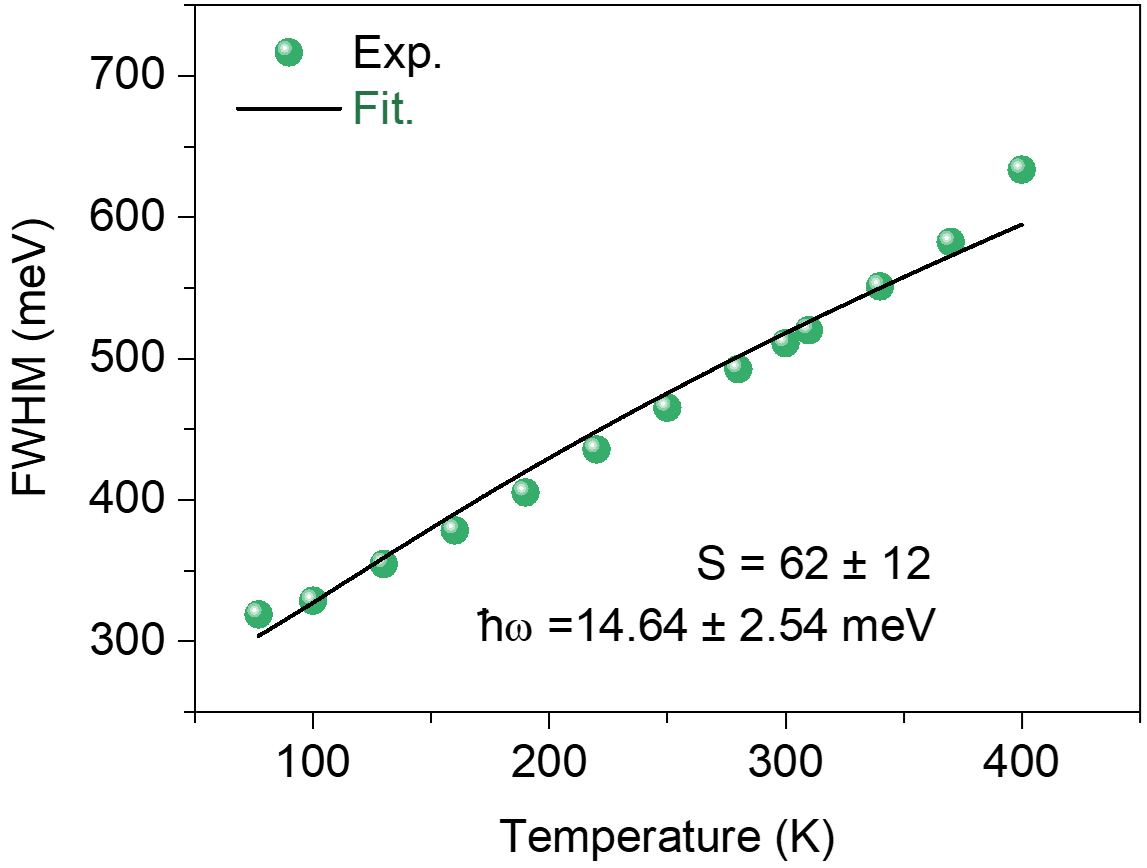


Figure S16. FWHM of Tl-bound exciton emission (510 nm) as a function of temperature. The fitted Huang-Rhys factor and phonon frequency are listed in the figure.


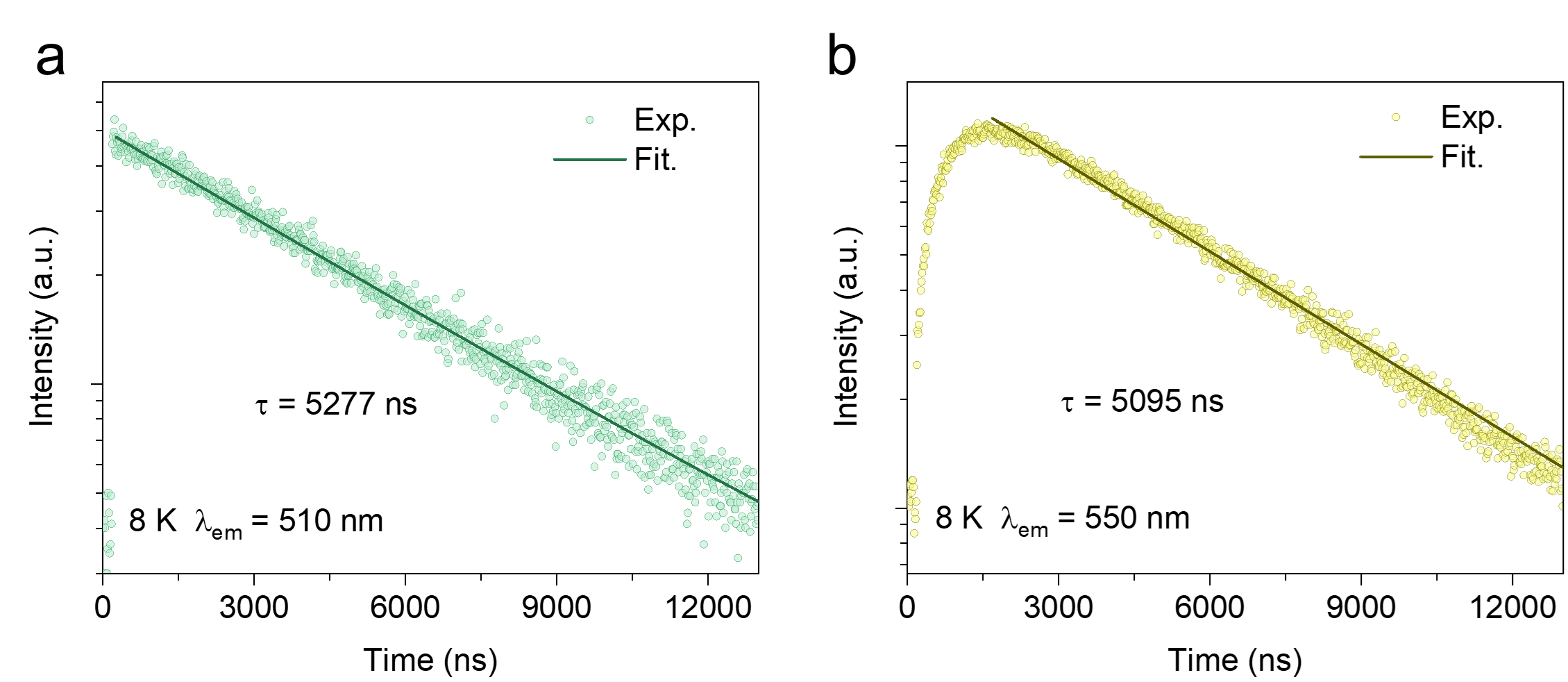


Figure S17. a, b, PL decay profile of emission at 510 nm (a) and emission at 550 nm (b) in Cs_3_Cu_2_I_5_:Tl single crystal at 8 K.


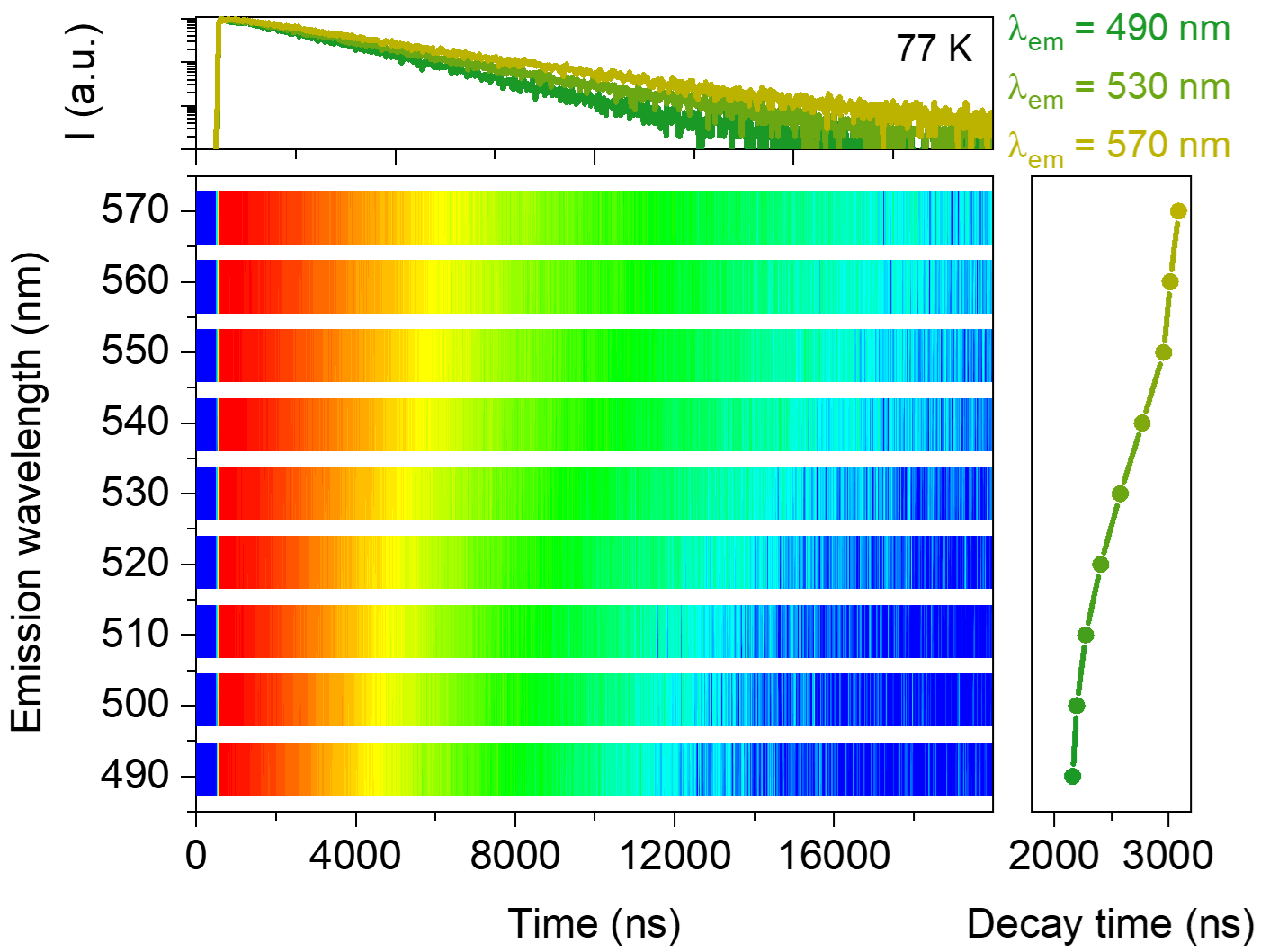


Figure S18. PL decay profile of emission from 490 to 570 nm under 330 nm excitation at 77 K. The upper figure shows the feature of single-exponential fitting. The derived results are shown in the right figure.

**Thermoluminescence**

Thermoluminescence (TL) glove curve measurement was performed with the custom-made thermoluminescence spectrometer. The samples with 4 mm × 4 mm × 3 mm were irradiated under X-ray (Au target, 50 kV, 75 μA) for 15 min at 77 K. Then the samples were heated from 77 K to 480 K with a heating rate of 0.1 K/s.

**
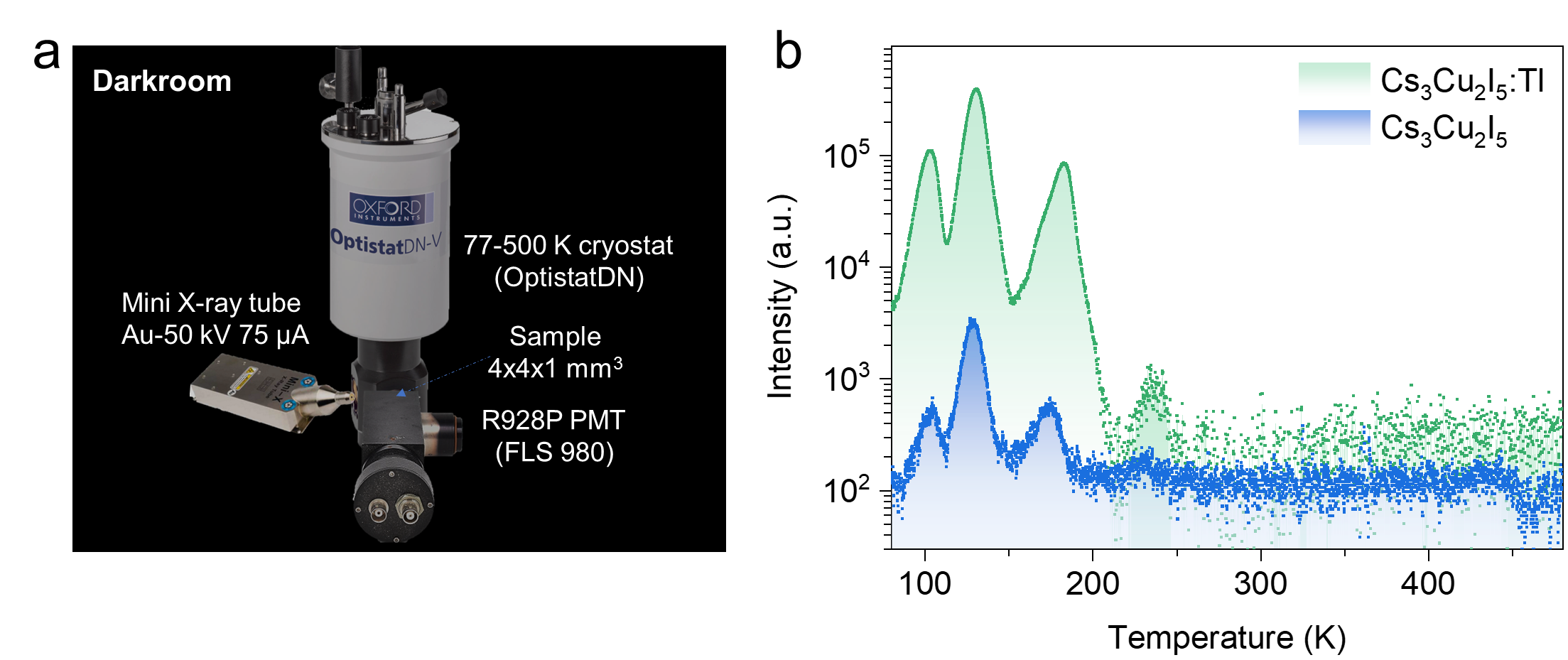
**

Figure S19. a, Schematic diagram of the custom-made TL spectrometer. b, TL glow curves of Cs_3_Cu_2_I_5_ and Cs_3_Cu_2_I_5_:Tl single crystals.

Particle detection and discrimination

Figure S20. Light yields of Cs_3_Cu_2_I_5_:Tl crystals under 662 keV gamma ray and 5.49 MeV alpha ray.


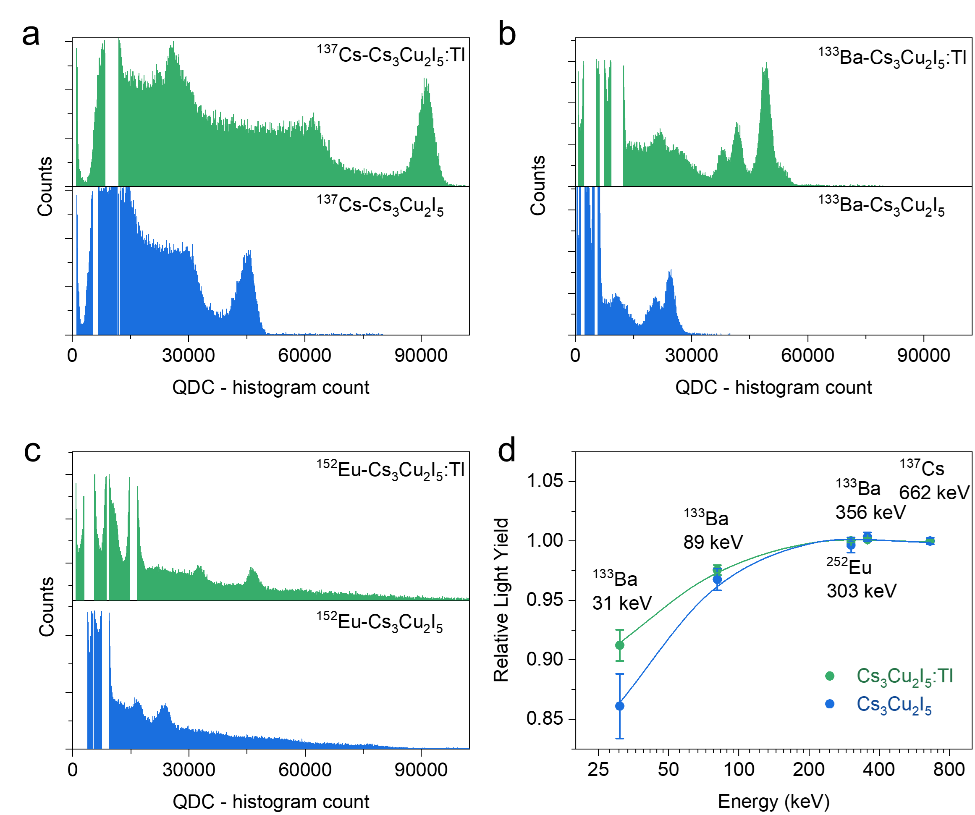


Figure S21 a, b, c, Pulse height spectra of Cs_3_Cu_2_I_5_ and Cs_3_Cu_2_I_5_:Tl under ^137^Cs (a), ^133^Ba (b), and ^152^Eu (c) γ-ray excitation. d, Relative light yields of Cs_3_Cu_2_I_5_ and Cs_3_Cu_2_I_5_:Tl at different energies.


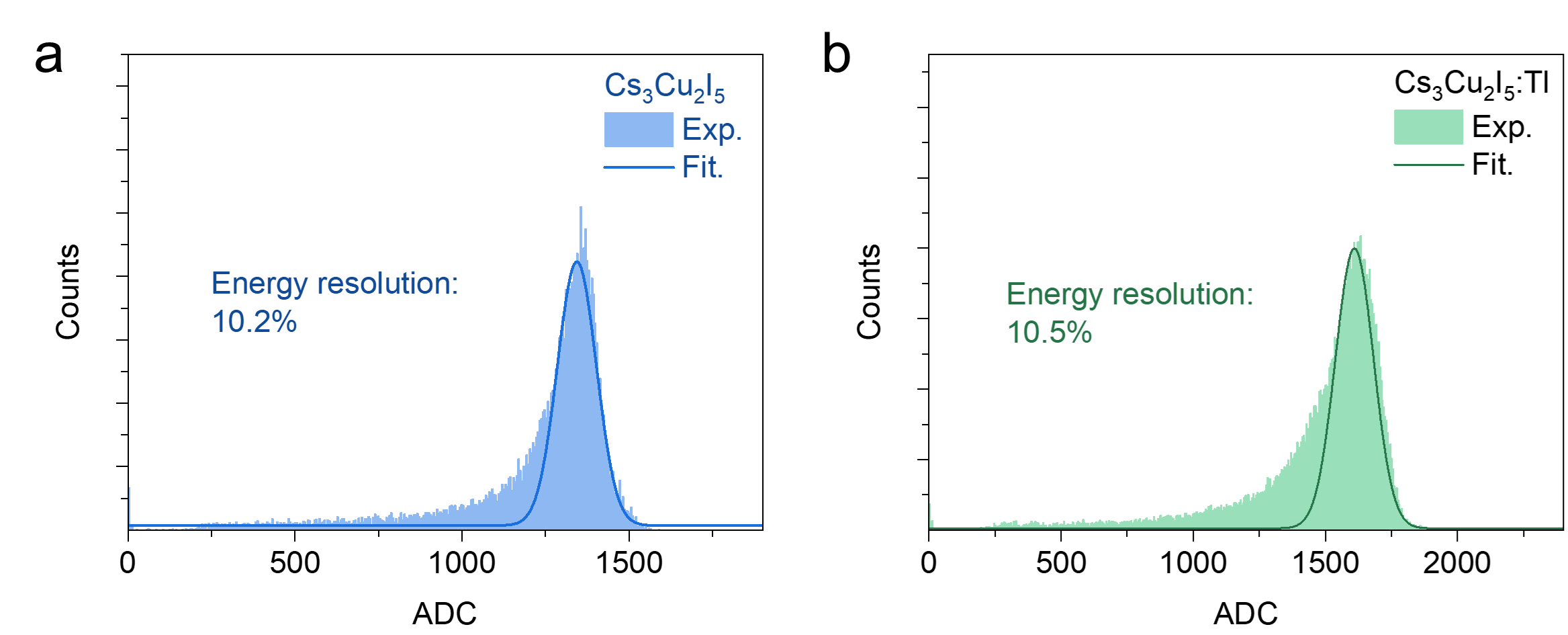


Figure S22. a, b, Pulse height spectra of Cs_3_Cu_2_I_5_ (a) and Cs_3_Cu_2_I_5_:Tl (b) under ^241^Am excitation acquired using Hamamatsu a R6233-100 PMT.


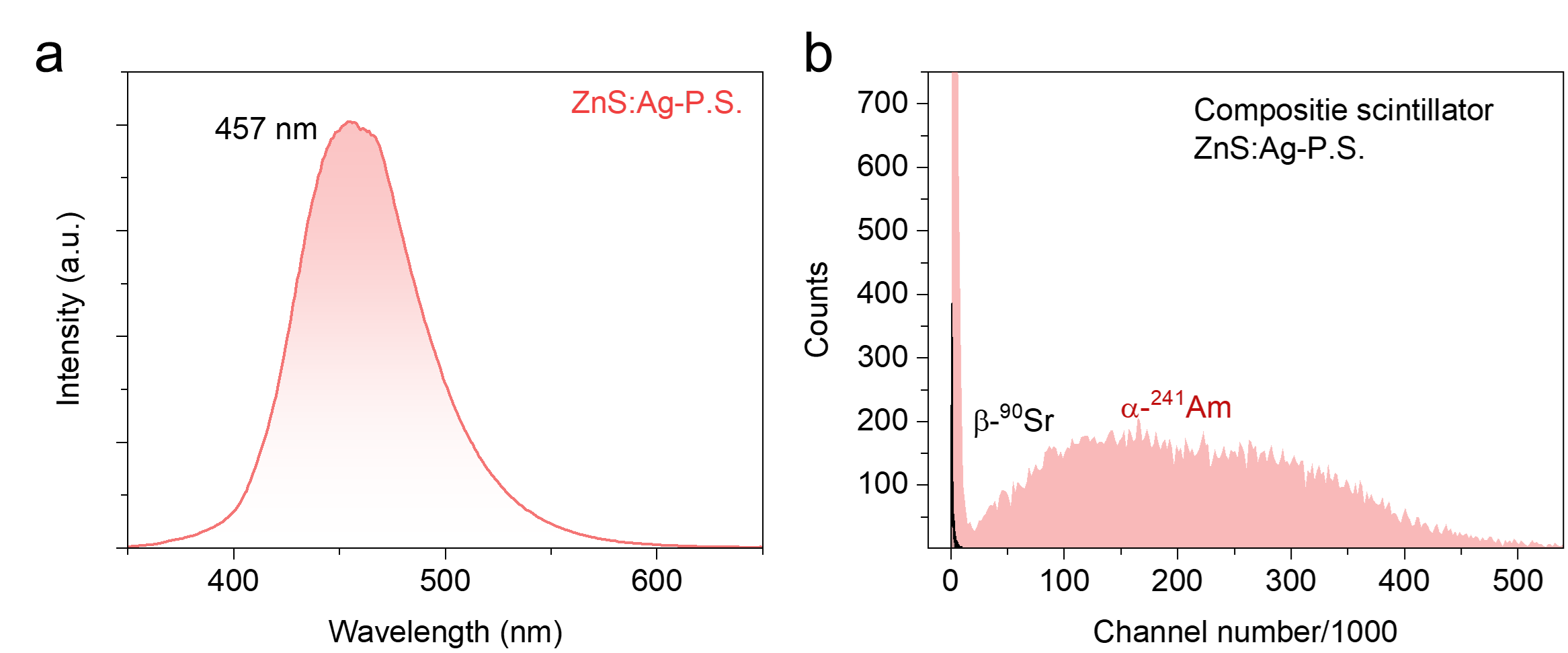


Figure S23. a, b, Emission spectra under X-ray excitation (a), and pulse height spectra under ^241^Am and ^90^Sr excitation (b) of composite detector based on ZnS:Ag and plastic scintillator.


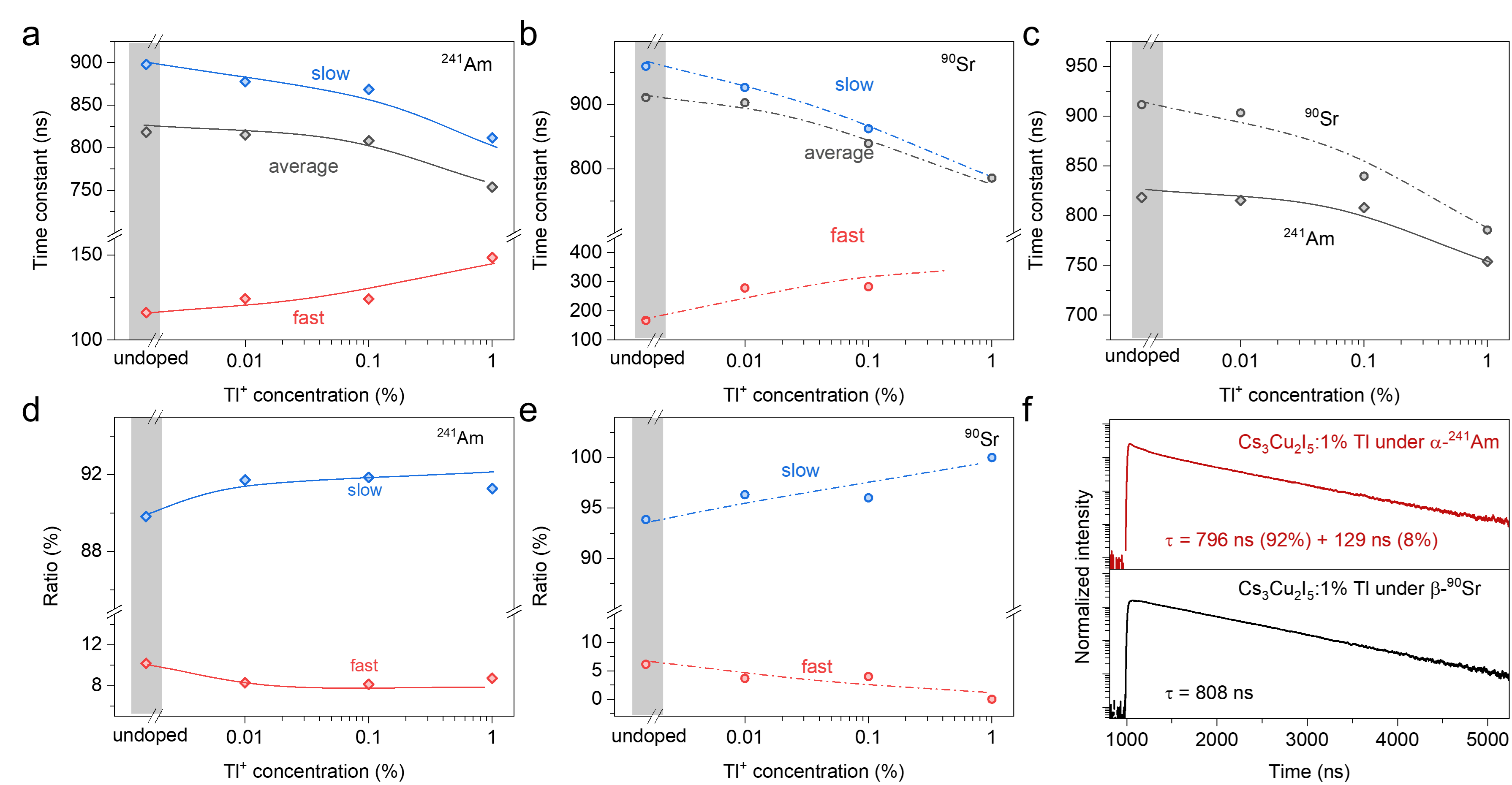


Figure S24. Scintillation decay evolution as a function of Tl concentration. a, d, Time constants (a) and ratios (d) of the fast and slow components under ^241^Am excitation. b, e, Time constants (b) and ratios (e) of the fast and slow components under ^90^Sr excitation. c, Averaged scintillation decay constants of Cs_3_Cu_2_I_5_:Tl. f, Scintillation decay profiles of Cs_3_Cu_2_I_5_:1%Tl under ^241^Am and ^90^Sr sources excitation.


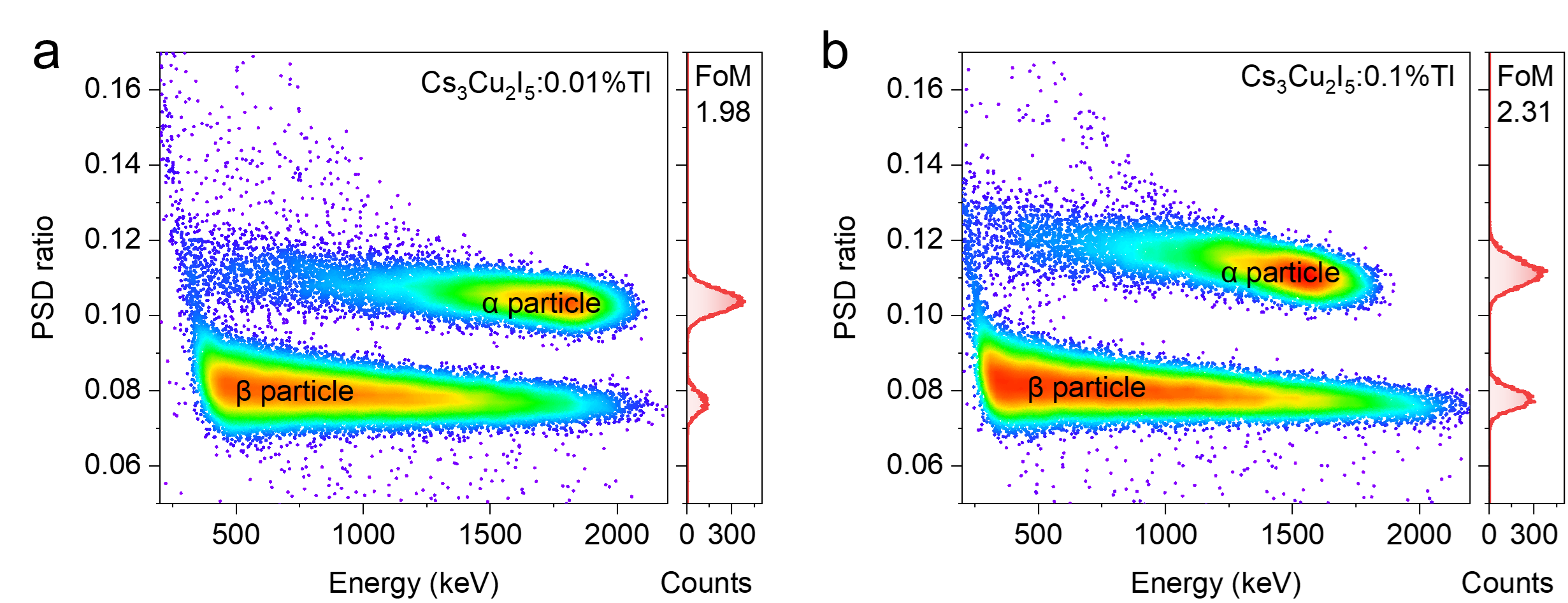


Figure S25. a, b, PSD scatter plots and spectra of Cs_3_Cu_2_I_5_:0.01%Tl (a) and Cs_3_Cu_2_I_5_:0.1%Tl (b) under the excitation of ^241^Am and ^90^Sr sources.

**^220^Rn loading and measurement:**

A ^232^Th diffusion source (1.2 × 10^3^ Bq) was used to produce ^220^Rn, which was then loaded into a 70 ml gas-wash bottle using a pump. The bottle loaded with ^220^Rn was placed upside-down on a Cs_3_Cu_2_I_5_:1%Tl crystal and coupled with a PMT (HZC PHOTONICS XP3240) for testing. The PMT was operated at -700V and the signal was recorded directly with a CAEN DT5751 digitizer for 3 hours. To serve as environmental background testing, an air-filled gas-wash bottle was used when measuring radon. This control setup served to establish a comparative context for evaluating the measured radon values.


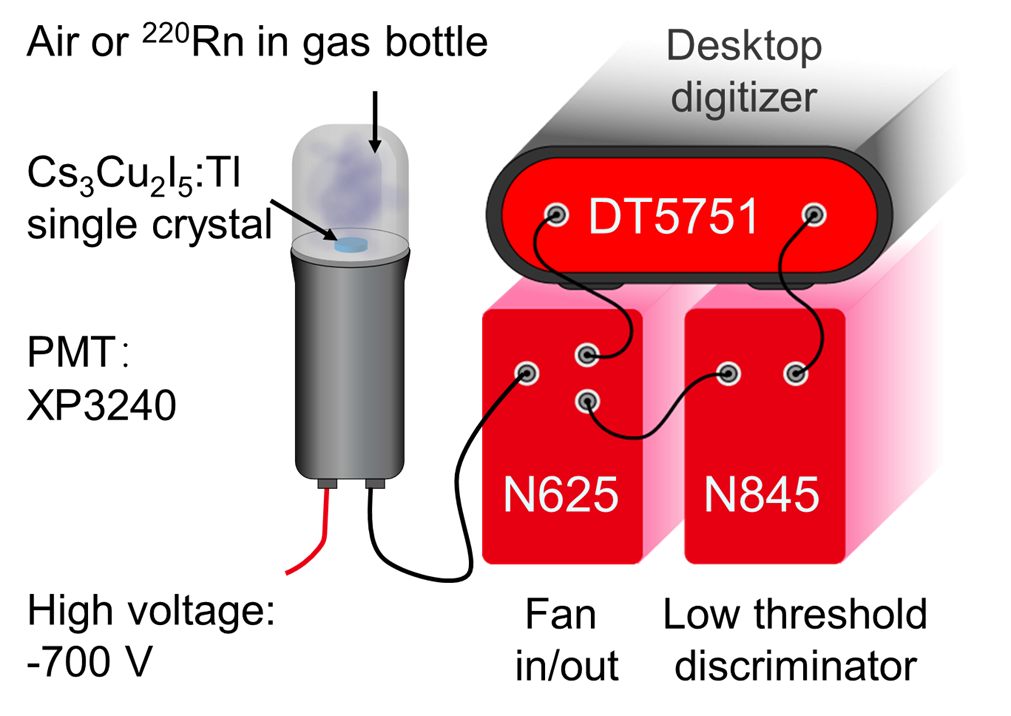


**Figure S26.** Schematic diagram of the preliminary ^220^Rn measurement system.

As compared to widely used scintillation cells (flask coated by scintillator, such as ZnS:Ag, et al.) and liquid scintillation devices (mixture of an organic solvent and flour, such as PPO-loaded DIN, et al.), the device offers a compact structure and lower usage cost.^18, 19, 20^ The time gate parameters used for the PSD were the same as the tests conducted under the excitation of ^241^Am and ^90^Sr sources.

The environmental test result of a gas wash bottle filled with natural air wherein 23 α events were detected during a sampling time of 3 hours, indicating the presence of α emitters in the air, building, or bottle material. The extra α events detected clearly originated from the ^220^Rn samples loaded into the bottle.

Comparation of α/β discrimination scintillators

Table S6. The physical and scintillation properties of 0D Cs_3_Cu_2_I_5_:Tl single crystal, conventional, and some newly developed solid scintillators for α/β discrimination application.

| Scintillators | Cs_3_Cu_2_I_5_:Tl single crystal | ZnS:Ag composition | CsI:Na single crystal | Plastic scintillator |
| --- | --- | --- | --- | --- |
| Density (g/cm^3^) | **4.5** | 4.1, ~1 | 4.5 | ~1 |
| Z_eff_ | **52.2** | N/A | 54 | ＜10 |
| Light yield at 662 keV (ph./MeV) | **83 000** | N/A | 41 000 | ~10 000 |
| Energy resolution at 662 keV (%) | **3.3** | N/A | 6.7 | N/A |
| PSD FoM | **2.64** | 1.92 | 3.0 (α/γ) | ~1 |
| Stability | **Stable** | Sensitive to temperature | Sensitive to humidity | Sensitive to temperature |
| Compact | **√** | × | √ | √ |
|  | **This work** | Ref.21 | Ref.22 | Ref.23 |

**Supplementary Reference**

1. Ronda, C.R., Srivastava, A.M. Scintillators. In: Ronda, CR (ed). *Luminescence: From Theory to Applications*, 2007, pp 105-132.

2. Zhang, F., et al. Blue–violet emitting K_2_CuCl_3_ compound: facile synthesis, photoluminescence and radioluminescence properties. *J Mater Sci*, **57**: 10260-10270 (2022).

3. Gao, W., et al. One-dimensional all-inorganic K_2_CuBr_3_ with violet emission as efficient X-ray scintillators. *ACS Applied Electronic Materials*, **2**: 2242-2249 (2020).

4. Zhao, X., et al. All-inorganic copper halide as a stable and self-absorption-free X-ray scintillator. *J Phys Chem Lett*, **11**: 1873-1880 (2020).

5. Cheng, S., et al. Non-hygroscopic, self-Absorption free, and efficient 1D CsCu_2_I_3_ perovskite single crystal for radiation detection. *ACS Appl Mater Interfaces*, **13**: 12198-12202 (2021).

6. Ma, Z., et al. Stable yellow light-emitting devices based on ternary copper halides with broadband emissive self-trapped excitons. *ACS Nano*, **14**: 4475-4486 (2020).

7. Niu, X., et al. Highly efficient blue emissive copper halide Cs_5_Cu_3_Cl_6_I_2_ scintillators for X-ray detection and imaging. *Ceram Int*, **48**: 30788-30796 (2022).

8. Li, J., et al. A highly efficient and stable blue-emitting Cs_5_Cu_3_Cl_6_I_2_ with a 1D chain structure. *Adv Mater*, **32**: e2002945 (2020).

9. Lian, L., et al. Highly luminescent zero-dimensional organic copper halides for X-ray scintillation. *J Phys Chem Lett*, **12**: 6919-6926 (2021).

10. Zhou, Q., et al. Highly efficient copper halide scintillators for high-performance and dynamic X-ray imaging. *Nanoscale*, **13**: 19894-19902 (2021).

11. Cheng, S., et al. Zero-dimensional Cs_3_Cu_2_I_5_ perovskite single crystal as sensitive X-ray and γ-ray scintillator. *Phys Status Solidi Rapid Res Lett*, **14**: 2000374 (2020).

12. Cheng, S., et al. Ultrabright and highly efficient all‐inorganic zero‐dimensional perovskite scintillators. *Adv Opt Mater*, **9**: 2100460 (2021).

13. Han, K., Jin, J., Su, B., Qiao, J., Xia, Z. Promoting single channel photon emission in copper(I) halide clusters for X-Ray detection. *Adv Opt Mater*, **10**: 2200865 (2022).

14. Xu, T., et al. Lead-free zero-dimensional organic-copper(I) halides as stable and sensitive X-ray scintillators. *ACS Appl Mater Interfaces*, **14**: 14157-14164 (2022).

15. Wojakowska, A., Górniak, A., Kuznetsov, A.Y., Wojakowski, A., Josiak, J. Phase Diagram of the System Copper(I) Iodide + Cesium Iodide. *Journal of Chemical & Engineering Data*, **48**: 468-471 (2003).

16. Doty, F.P., et al. Structure and properties of lanthanide halides. *Penetrating Radiation Systems and Applications VIII*; 2007.

17. Mao, R., Zhang, L., Zhu, R.-Y. Optical and scintillation properties of inorganic scintillators in high energy physics. *IEEE Trans Nucl Sci*, **55**: 2425-2431 (2008).

18. Li, J., et al. Novel liquid scintillator radon detector. *Radiation Detection Technology and Methods*, **6**: 294-301 (2022).

19. Feng, X.-g., He, Q.-g., Wang, J.-c., Chen, J. A general method for optimizing the parameter of α/β discrimination in liquid scintillation counting. *Anal Methods*, **6**: 115-119 (2014).

20. Abdalla, A.M., et al. Radon detection using alpha scintillation KACST cell. *Nucl Instrum Methods Phys Res A*, **922**: 84-90 (2019).

21. Ifergan, Y., et al. Development of a thin, double-sided alpha/beta detector for surface-contamination measurement. *IEEE Trans Nucl Sci*, **63**: 634-638 (2016).

22. Winyard, R.A., Lutkin, J.E., McBeth, G.W. Pulse shape discrimination in inorganic and organic scintillators. I. *Nucl Instrum Methods*, **95**: 141-153 (1971).

23. Morishita, Y., Di Fulvio, A., Clarke, S.D., Kearfott, K.J., Pozzi, S.A. Organic scintillator-based alpha/beta detector for radiological decontamination. *Nucl Instrum Methods Phys Res A*, **935**: 207-213 (2019).
